# Intron Retention in the 5′UTR of the Novel ZIF2 Transporter Enhances Translation to Promote Zinc Tolerance in *Arabidopsis*

**DOI:** 10.1371/journal.pgen.1004375

**Published:** 2014-05-15

**Authors:** Estelle Remy, Tânia R. Cabrito, Rita A. Batista, Mohamed A. M. Hussein, Miguel C. Teixeira, Alekos Athanasiadis, Isabel Sá-Correia, Paula Duque

**Affiliations:** 1 Instituto Gulbenkian de Ciência, Oeiras, Portugal; 2 Institute for Biotechnology and BioEngineering (IBB), Centre for Biological and Chemical Engineering, Department of Bioengineering, Instituto Superior Técnico, University of Lisbon, Lisbon, Portugal; Wageningen University, Netherlands

## Abstract

Root vacuolar sequestration is one of the best-conserved plant strategies to cope with heavy metal toxicity. Here we report that zinc (Zn) tolerance in *Arabidopsis* requires the action of a novel Major Facilitator Superfamily (MFS) transporter. We show that ZIF2 (Zinc-Induced Facilitator 2) localises primarily at the tonoplast of root cortical cells and is a functional transporter able to mediate Zn efflux when heterologously expressed in yeast. By affecting plant tissue partitioning of the metal ion, loss of *ZIF2* function exacerbates plant sensitivity to excess Zn, while its overexpression enhances Zn tolerance. The *ZIF2* gene is Zn-induced and an intron retention event in its 5′UTR generates two splice variants (*ZIF2.1* and *ZIF2.2*) encoding the same protein. Importantly, high Zn favours production of the longer *ZIF2.2* transcript, which compared to *ZIF2.1* confers greater Zn tolerance to transgenic plants by promoting higher root Zn immobilization. We show that the retained intron in the *ZIF2* 5′UTR enhances translation in a Zn-responsive manner, markedly promoting ZIF2 protein expression under excess Zn. Moreover, Zn regulation of translation driven by the *ZIF2.2* 5′UTR depends largely on a predicted stable stem loop immediately upstream of the start codon that is lost in the *ZIF2.1* 5′UTR. Collectively, our findings indicate that alternative splicing controls the levels of a Zn-responsive mRNA variant of the ZIF2 transporter to enhance plant tolerance to the metal ion.

## Author Summary

Alternative splicing, which generates multiple messenger RNAs (mRNAs) from the same gene, is a key posttranscriptional regulatory mechanism in higher eukaryotes whose functional relevance in plants remains poorly understood. The sequestration of metal ions inside the vacuole of root cells is an important strategy employed by plants to cope with heavy metal toxicity. Here, we describe a new vacuolar membrane transporter of the model plant *Arabidopsis thaliana*, ZIF2, that confers tolerance to zinc (Zn) by promoting root immobilisation of the metal ion and thus its exclusion from the aerial parts of the plant. The *ZIF2* gene is induced by exposure to excess Zn and undergoes alternative splicing, generating two mRNAs that differ solely in their non-coding regions and hence code for the same transporter. Interestingly, toxic Zn levels favour expression of the longer mRNA, which in turn confers higher plant tolerance to the metal. We show that the longer *ZIF2* non-coding region markedly promotes translation of the downstream coding sequence into protein in a Zn-responsive fashion. Thus, our results indicate that by regulating translation efficiency of the *ZIF2* mRNA, alternative splicing controls the amounts of the encoded membrane transporter and therefore plant Zn tolerance.

## Introduction

The transition metal zinc (Zn) is a micronutrient essential for optimal plant growth and development, serving as a catalytic co-factor or structural motif in numerous proteins required for basic cellular processes. However, its propensity to inactivate other crucial proteins also renders Zn a potentially toxic element that can adversely affect plant growth when present in excessive amounts [1]. To deal with these opposing effects and adjust to environmental fluctuations in Zn availability, plants developed a tightly controlled homeostatic network aimed at ensuring an adequate supply of the nutrient while preventing its toxic build-up at the cellular and whole-plant levels [2].

Zn is acquired from the soil as a divalent cation that once absorbed into the root epidermis moves mostly symplastically through the adjacent cell layers to the central stele. After active loading into the xylem vessels, Zn is translocated to the shoot via the transpiration stream and subsequently transferred to the phloem before allocation to aerial organs [3]. While plants adapt to zinc shortage supply prevalently by activating cellular Zn uptake particularly at the root-soil interface [4], cellular Zn detoxification mechanisms are primarily intended at restricting the accumulation of free cytosolic Zn^2+^, mainly through its extrusion to the apoplasm [5], chelation with specific ligands [6], [7] and/or vacuolar compartmentalisation [8]. At the whole-plant level, tolerance to excess Zn is achieved through the rearrangement of its tissue partitioning via enhanced Zn sequestration in leaves [9], whereas within the root both immobilisation in the outer cell layers [10] and exclusion from the epidermis [11] contribute to limit Zn entry into the root symplasm.

All steps in plant Zn homeostasis, from initial acquisition by the root to subsequent distribution among different tissues and subcellular trafficking, require the concerted action of multiple Zn membrane transport systems. Three groups of such carrier proteins have been primarily characterised in *Arabidopsis thaliana*. While members of the ZIP/IRT (Zinc-regulated transporter, Iron-regulated transporter-like Protein) family have been proposed to constitute the major cellular Zn uptake system [12]–[14], HMAs (Heavy–metal transporting ATPases) and MTPs (Metal Tolerance Proteins), which belong to the P_1B_-type ATPases and Cation Diffusion Facilitator (CDF) families, respectively, are essential for full Zn tolerance owing to their ability to drive either Zn whole-plant partitioning or organelle sequestration/exclusion [8]–[10], [15]–[19]. The recent implication of two novel *Arabidopsis* transporters, PCR2 (Plant Cadmium Resistance 2; [11]) and the Major Facilitator Superfamily (MFS) carrier ZIF1 (Zinc-Induced Facilitator 1; [20]), in Zn tolerance suggests that additional plant Zn transport components are still to be discovered.

On the fringe of the vast majority of plants that like *A. thaliana* typically possess basal Zn tolerance, a specialized flora displays naturally selected tolerance to extremely high Zn concentrations, being able to accumulate considerable Zn amounts in their aboveground parts without developing toxicity symptoms. This hyperaccumulation strategy relies primarily on efficient xylem loading and vacuolar sequestration in leaves, thus inducing a systemic Zn-deficiency status that stimulates root Zn uptake [21]. Cross-species transcriptomic profiling particularly in the hyperaccumulator *Arabidopsis halleri*, a close *A. thaliana* relative, has established that key Zn transporters, such as HMA4, MTP1, HMA3 and ZIP/IRTs, are not different but rather constitutively overexpressed in hyperaccumulators [22]–[24]. Considering not only differential expression between the two *Arabidopsis* species but also individual regulation by exogenous Zn, Becher et al. [23] provided a shortlist of 50 promising candidate genes for a role in Zn homeostasis that notably includes At2g48020 encoding a yet uncharacterised MFS carrier, which given the *Arabidopsis* ZIF1 role in zinc tolerance [20] raises particular interest.

MFS transporters are single-polypeptide secondary carriers using chemiosmotic gradients as an energy source and ubiquitous to all classes of organisms. With more than 120 predicted members, the MFS represents the second widest group of *Arabidopsis* transporters after the ATP-Binding Cassette (ABC) superfamily [25], [26]. Interestingly, according to the current genome annotation (TAIR V10; www.arabidopsis.org), 34 MFS genes undergo alternative splicing, a key generator of proteomic diversity and functional complexity in higher eukaryotes. Despite the fact that over 60% of the *Arabidopsis* multi-exon genes are currently estimated to produce more than one transcript via this mRNA processing mechanism [27], its biological significance in plants is poorly understood, having been uncovered for only a dozen genes [28], [29].

Here, we report that the MFS transporter encoded by the At2g48020 gene, which we named *ZIF2*, sustains plant Zn tolerance in *A. thaliana* by mediating root vacuolar sequestration of the metal, thus preventing its translocation to the shoot. Importantly, we found that elevated Zn levels favour expression of a *ZIF2* splice variant whose longer 5′ untranslated region (UTR) enhances the translation efficiency of the ZIF2 transporter and hence plant Zn tolerance.

## Results

### The *ZIF2* Promoter Is Ubiquitously Active in *Arabidopsis*

**Figure 1:**
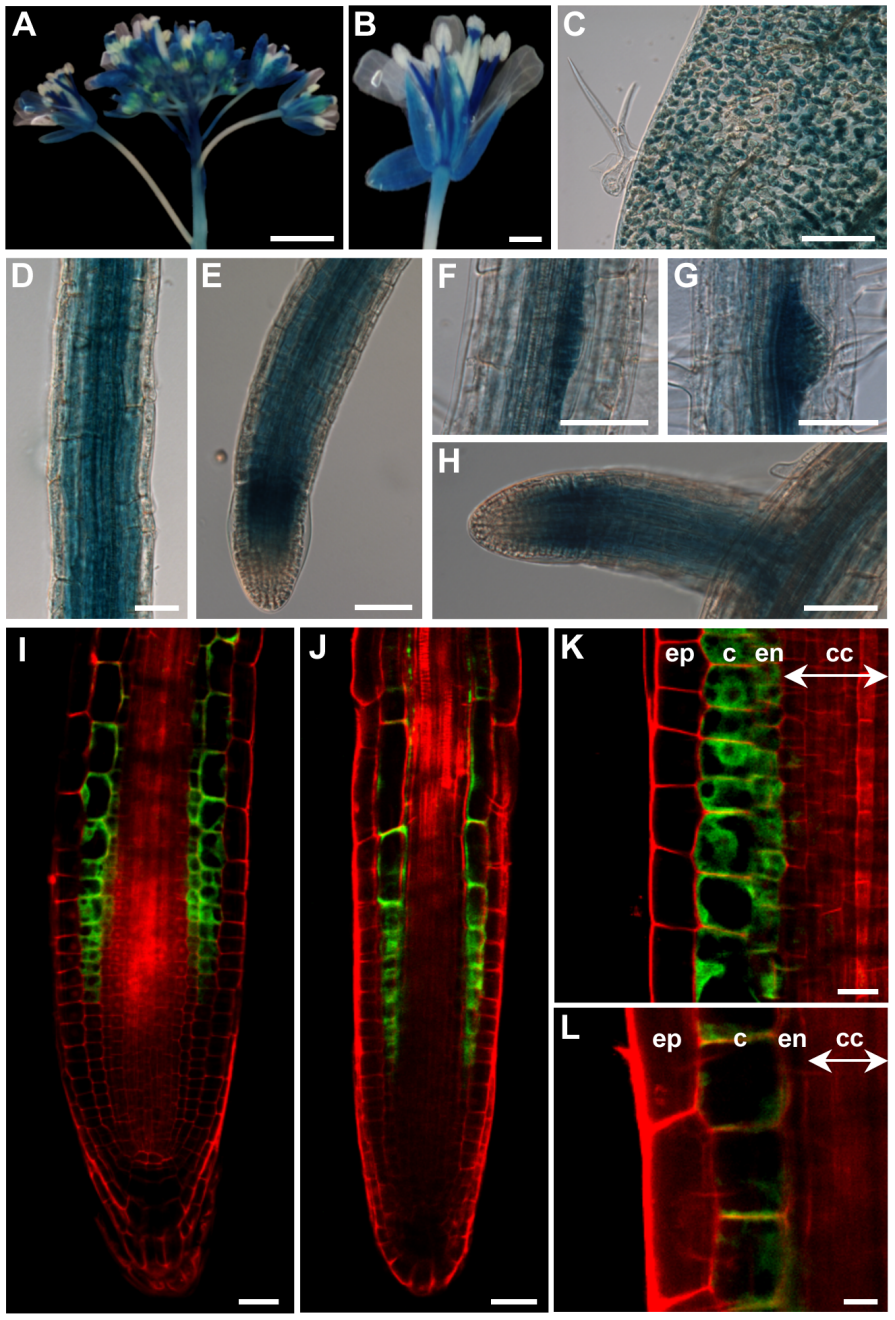
The *ZIF2* promoter is active in most *Arabidopsis* tissues. (A–H) Differential interference contrast microscopy images of GUS-stained transgenic plants carrying the *ProZIF2:GFP-GUS* reporter construct. *ZIF2* promoter activity in floral buds (A), a mature flower (B), a young leaf (C), the primary root (D), the primary root tip (E), a lateral root primordium at stage V (F) and stage VII (G) and a mature lateral root (H). Scale bars, 1 mm (A), 200 µm (B, C), or 50 µm (D–H). (I–L) Confocal laser scanning microscopy images of transgenic root tissues carrying the *ProZIF2:GFP-GUS* reporter construct. *ZIF2* promoter activity in the primary root (I, K) and a mature lateral root (J, L). ep, epidermis; c, cortex; en, endodermis; cc, central cylinder. Cell walls were stained with propidium iodide. The GFP and propidium iodide signals are visualized by green and red coloration, respectively. Scale bars, 25 µm (I, J), or 10 µm (K, L).

To initiate the characterisation of the *ZIF2* gene (At2g48020), we examined its organ- and tissue-specific expression pattern by means of reporter gene experiments. Staining of transgenic *Arabidopsis* lines stably expressing β-glucuronidase (GUS) under the control of the *ZIF2* promoter revealed that the latter is active in most plant organs (Figure 1A–H). In particular, *ZIF2* was highly expressed at all stages of floral development (Figure 1A), with flower buds displaying exclusive staining of the style, stamen filaments and sepals (Figure 1B). Promoter activity was also detected in leaf mesophyll cells (Figure 1C) and strongly throughout the root system (Figure 1D–H). Although homogenous GUS coloration was observed all along the primary root (PR) (Figure 1D), the *ZIF2* promoter was particularly active at the root tip (Figure 1E). We detected very intense GUS staining in lateral root (LR) primordia (Figure 1F, G), but *ZIF2* was also abundantly expressed in elongating LRs (Figure 1H).

We further examined the precise root tissue distribution of *ZIF2* promoter activity using transgenic plants expressing the green fluorescent protein (GFP) under the control of the native *ZIF2* promoter (Figure 1I–L). At the PR tip, *ZIF2* promoter activity was confined to the endodermal and cortical cell layers of the elongation and transition zones, while absent from the apical meristem, the quiescent centre, columella cells and the lateral root cap (Figure 1I). Similarly, in the mature portion of the PR, the GFP signal was detected in both the endodermis and the cortex (Figure 1K). A slightly different layer-specific pattern was observed in LRs, with *ZIF2* expression appearing largely restricted to the cortex, particularly at the tip (Figure 1J, L). *ZIF2* promoter activity in flowers and leaves was insufficient to allow detection of the GFP signal. Collectively, the above results suggest that ZIF2 exerts a prominent role in the root system, particularly in the PR.

### High Zn Induces *ZIF2* Expression and Promotes an Intron Retention Event in Its 5′UTR

**Figure 2:**
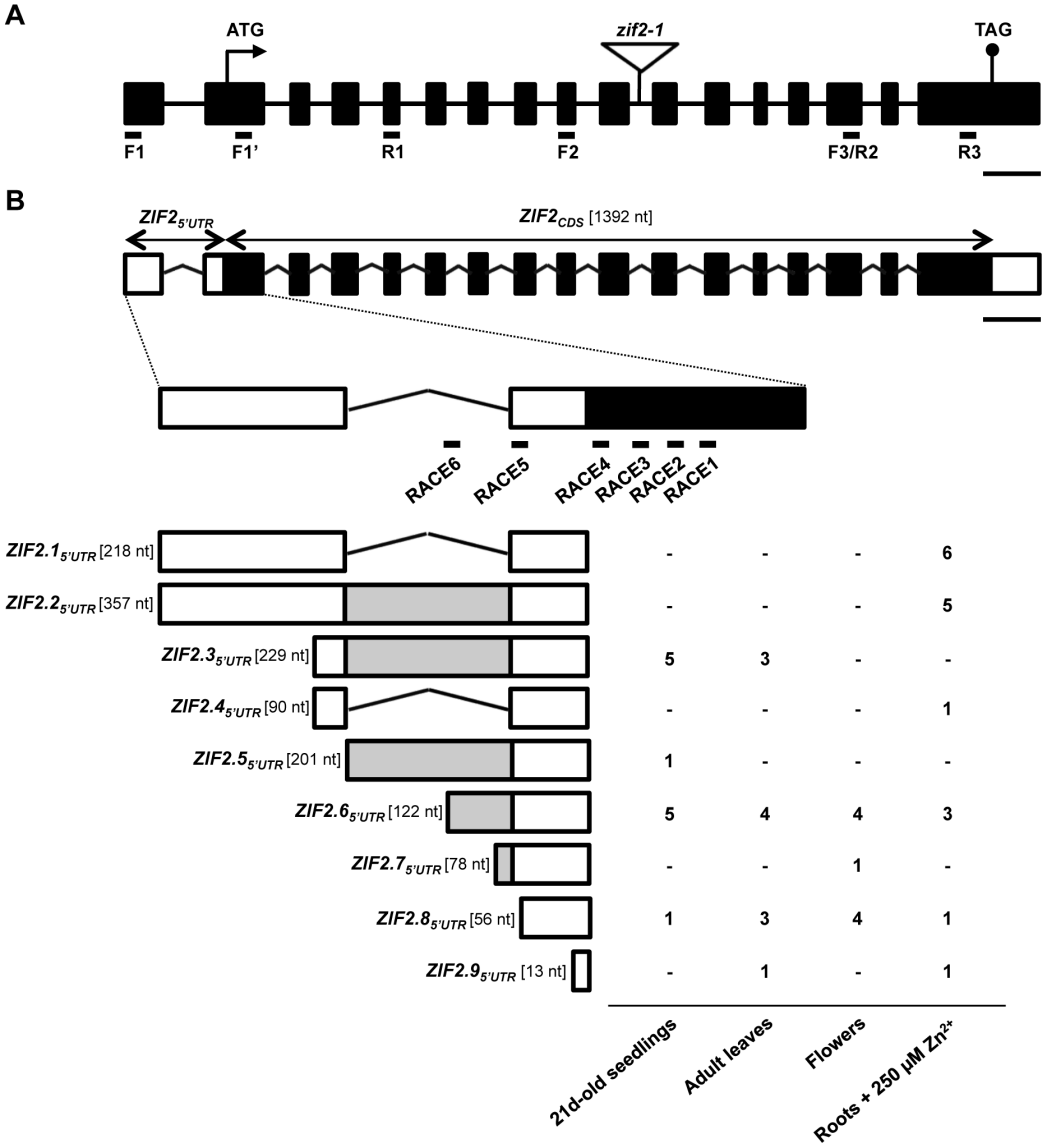
The *Arabidopsis ZIF2* gene encodes multiple transcripts. (A) Exon/intron organization of the *ZIF2* gene and T-DNA insertion site in the *zif2-1* mutant. Boxes and lines between boxes denote exons and introns, respectively. The triangle depicts the site of the T-DNA insertion. F1, F1′, F2, F3, R1, R2, and R3 indicate the location of the primers used to detect *ZIF2* expression in Figures 3A, 3C, 7A, S1 and S4. Scale bar, 200 bp. (B) Structure of the alternative *ZIF2* transcripts. The coding sequence (CDS) is shown in black and the UTRs in white. Grey boxes indicate portions of the 5′UTR overlapping with the retained intron. RACE1 to RACE6 indicate the location of the gene-specific primers used in the 5′RLM-RACE experiment. Occurrence of each *ZIF2* transcript was determined according to its detection (number of corresponding 5′RLM-RACE clones) or not (-) by RLM-5′RACE in the indicated tissue. Scale bar, 200 nt.

According to the current genome annotation (TAIR V10), the *Arabidopsis ZIF2* gene contains 17 exons (Figure 2A) and generates two distinct mRNAs that differ solely in their 5′UTRs. In order to verify the accuracy of these predictions and examine the tissue-specific distribution of the *ZIF2* transcripts, we performed RNA Ligase-Mediated-5′ Rapid Amplification of cDNA Ends (RLM-5′RACE) in different *Arabidopsis* vegetative and reproductive tissues, i.e. in seedlings, adult leaves and flowers. Given the high *ZIF2* promoter activity detected throughout the root system (see Figure 1) and the differential expression of the *ZIF2* orthologue reported in the Zn hyperaccumulator *A. halleri* [23], we also performed RLM-5′RACE in roots of seedlings exposed to Zn toxicity. As shown in Figure 2B, we were able to identify nine alternative *ZIF2* 5′UTRs and named the corresponding transcripts *ZIF2.1* (At2g48020.1 according to TAIR V10) to *ZIF2.9*. A common start codon is predicted for all *ZIF2* transcripts, and we verified the absence of an in-frame ATG or alternate start codon in the nine 5′UTRs as well as of any alterations in the coding sequence. Hence, all *ZIF2* transcripts are predicted to encode the same 53-kDa full-size transporter, displaying the typical MFS transporter signature motif that includes two transmembrane domains, each consisting of six membrane-spanning segments, delimiting a central hydrophilic loop [25]. Distribution of the *ZIF2* transcripts was found to exhibit tissue specificity (Figure 2B). Interestingly, the *ZIF2.1* and *ZIF2.2* splice variants, which result from retention of a 139-nt intron in the 5′UTR, appeared to be predominantly expressed in roots challenged with high Zn concentrations, hinting at a role for this intron retention event in roots exposed to excess Zn.

**Figure 3:**
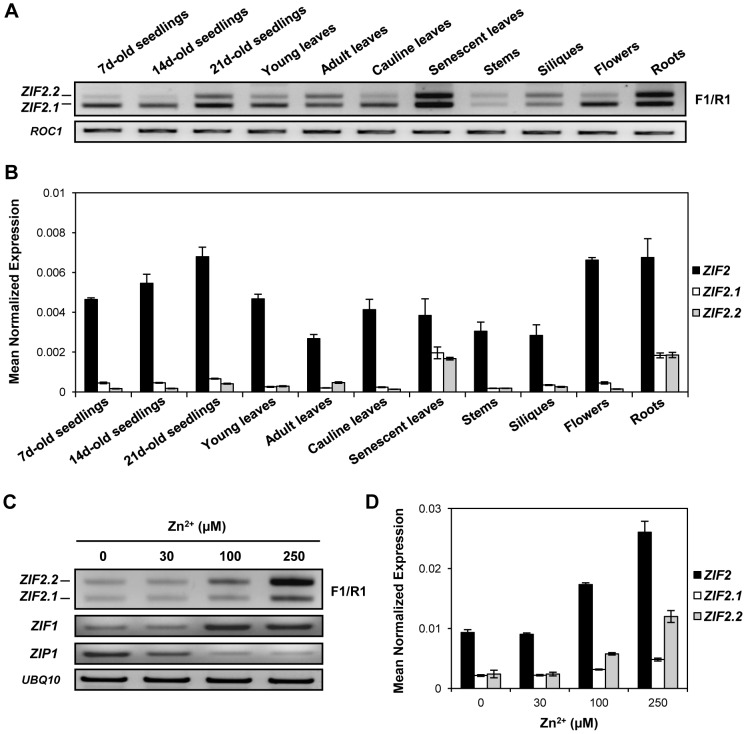
The expression and splicing patterns of the *Arabidopsis ZIF2* gene are Zn-regulated. (A) RT-PCR profile of *ZIF2.1 and ZIF2.2* expression in different wild-type (Col-0) tissues. The location of the F1 and R1 primers used is shown in Figure 2A. Expression of the *ROC1* gene was used as a loading control. Results are representative of three independent experiments. (B) Real-time RT-PCR analysis of total *ZIF2* expression as well as of *ZIF2.1* and *ZIF2.2* transcript levels in different wild-type (Col-0) tissues, using *UBQ10* as a reference gene. Results are from two independent experiments and values represent means ± SE (*n* = 4). (C) RT-PCR profile of *ZIF2.1* and *ZIF2.2* expression in roots of 7-d old wild-type (Col-0) seedlings challenged for 48 h with various Zn supplies. The location of the F1 and R1 primers used is shown in Figure 2A. Expression of the *ZIF1* and *ZIP1* or *UBQ10* genes is shown as plant metal status or loading controls, respectively. Results are representative of three independent experiments. (D) Real-time RT-PCR analysis of total *ZIF2* expression as well as of *ZIF2.1* and *ZIF2.2* transcript levels in roots of 7-d old wild-type (Col-0) seedlings challenged for 48 h with various Zn supplies, using *UBQ10* as a reference gene. Results are from two independent experiments and values represent means ± SE (*n* = 4).

We then examined the tissue-specific distribution of the *ZIF2.1 and ZIF2.2* splice variants by RT-PCR (Figure 3A, B). Consistent with the promoter activity profile shown in Figure 1, RT-qPCR analysis revealed that *ZIF2* is expressed throughout plant development, particularly in roots and flowers of mature plants, and to a lesser extent in young seedlings, while lower transcript levels were detected in other aboveground tissues such as developing siliques, stems or leaves (Figure 3B). By contrast, the Z*IF2.1* and *ZIF2.2* transcripts were detected at very low levels in all tissues analysed, except in roots of mature plants where the expression level of the two splice variants combined was found to represent more than half of total *ZIF2* expression. *ZIF2.1* and *ZIF2.2* expression was also markedly induced during leaf senescence (Figure 3A, B).

We next asked whether *ZIF2* expression would be responsive to exogenous Zn in *A. thaliana* roots. Both total *ZIF2* expression and the equivalent amounts of the *ZIF2.1* and *ZIF2.2* splice variants detected in root tissues under control Zn conditions (30 µM) appeared unchanged by Zn deficiency (Figure 3C, D). However, concomitant with a significant induction of *ZIF2* expression, a clear shift in the *ZIF2* splicing pattern occurred after at least 48 h of challenge with high Zn (Figure 3C, D; Figure S1). Indeed, root *ZIF2.2* expression was markedly up-regulated in a concentration-dependent manner following exposure to Zn excess, even as slight as 100 µM, while a considerable increase in *ZIF2.1* steady-state levels was also observed but of lower magnitude and only under toxic Zn excess (250 µM). Thus, both total expression of the *ZIF2* gene and alternative splicing of its precursor mRNA (pre-mRNA) are regulated by the plant's Zn status, with high levels of the metal ion promoting retention of the 5′UTR intron.

### *ZIF2* Encodes a Functional Tonoplast-Localised Transporter

**Figure 4:**
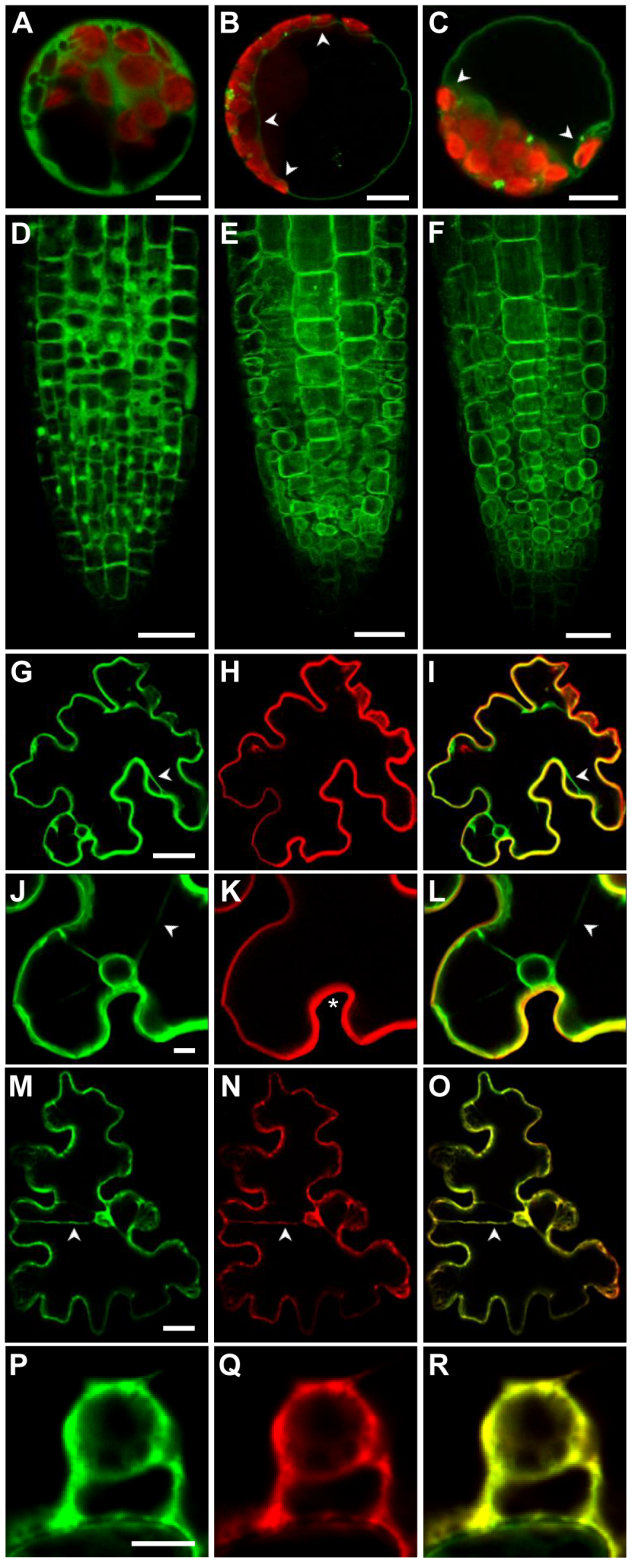
The *Arabidopsis* ZIF2 transporter localises at the tonoplast. (A–C) Confocal laser scanning microscopy images of *Arabidopsis* wild-type mesophyll protoplasts transiently expressing either YFP alone (A) or the ZIF2.1-YFP (B) or ZIF2.2-YFP (C) fusions under the control of the 35S promoter. Arrowheads point to the YFP signal on the inner side of the chloroplasts and the nucleus. The YFP and chloroplast autofluorescence signals are visualized by green and red coloration, respectively. Scale bars, 10 µm. (D–F) Confocal laser scanning microscopy images of transgenic *Arabidopsis* root tips expressing either YFP alone (D) or the ZIF2.1-YFP (E) or ZIF2.2-YFP (F) fusions under the control of the 35S promoter. The YFP signal is visualized by green coloration. Scale bars, 50 µm. (G–R) Confocal laser scanning microscopy images of tobacco leaf epidermal cells transiently co-expressing the ZIF2.2-GFP fusion (G, J, M, P) with the plasma membrane marker PIP2A-mCherry (H, K) or the tonoplast marker g-TIP-mCherry (N, Q) under the control of the 35S promoter. Merged images of whole-cell views (I, O) or nucleus close-ups (L, R) are shown. Arrowheads point to transvacuolar strands and asterisks indicate fluorescence signals approaching the nucleus only on the side facing the exterior of the cell. The GFP and mCherry signals are visualized by green and red coloration, respectively. Scale bars, 20 µm.

To determine the subcellular localisation of the protein encoded by the *ZIF2* transcripts, we generated C-terminal yellow fluorescent protein (YFP) or GFP fusions of each cDNA under the control of the 35S promoter. Confocal microscopy analysis of the YFP signal upon transient or stable expression in *Arabidopsis* protoplasts (Figure 4A–C) or root apices (Figure 4D–F), respectively, suggested that ZIF2 localises to the vacuolar membrane. In co-localisation experiments in tobacco leaf epidermal cells using specific tonoplast and plasma-membrane RFP markers [30], the ZIF2.2-GFP fusion protein co-localised exclusively with the tonoplast marker, whereas it did not match the distribution of the plasma membrane marker (Figure 4G–R), confirming that the ZIF2 transporter is primarily targeted to the tonoplast of plant cells.

**Figure 5:**
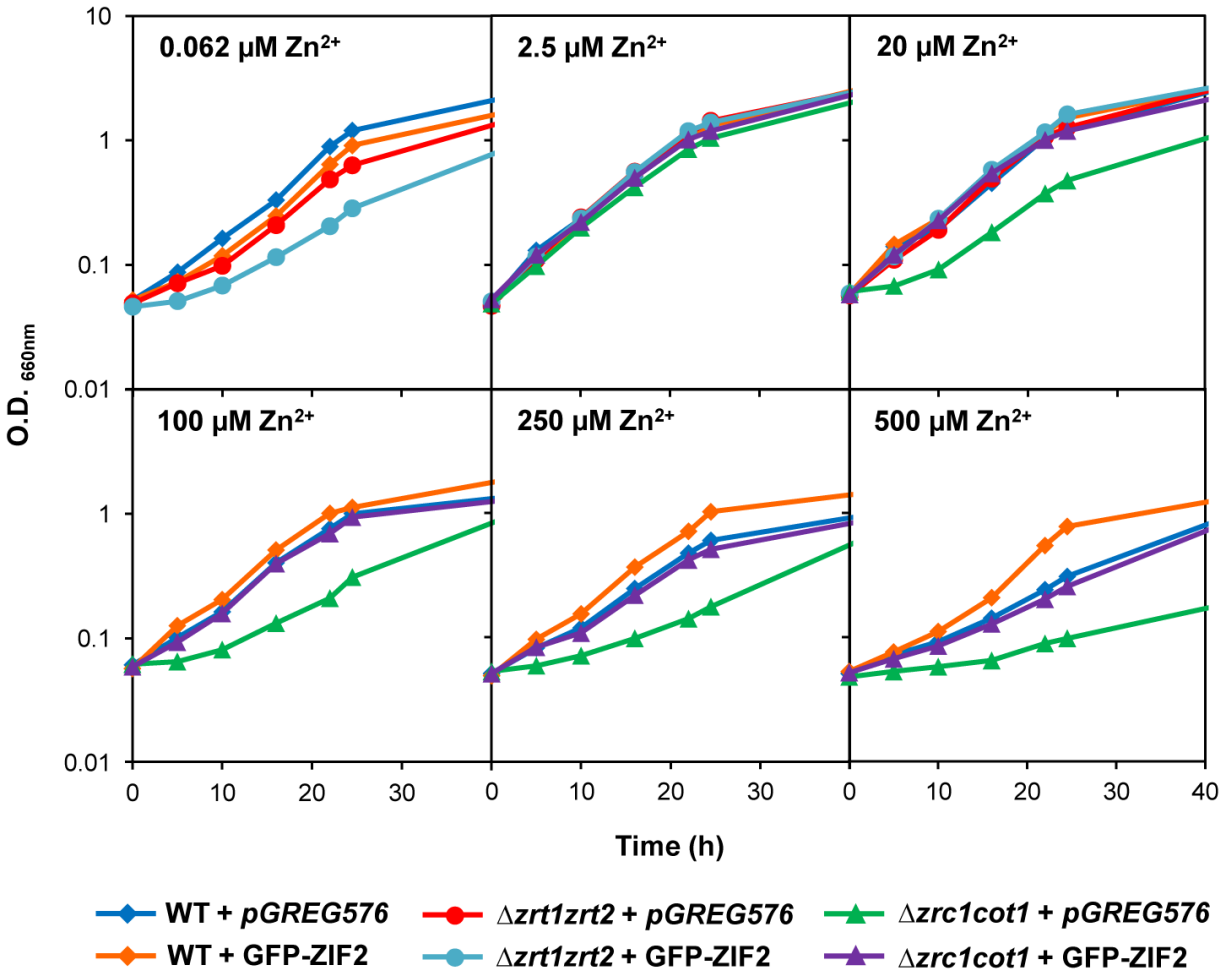
The *Arabidopsis* ZIF2 transporter confers Zn resistance to *S. cerevisiae*. Comparison of the growth curves of non-adapted wild-type, *Δzrt1zrt2* and *Δzrc1cot1* mutant yeast cells, harbouring either the cloning vector *pGREG576* or the GFP-ZIF2-encoding *pGREG576_ZIF2* plasmid, in Zn-free liquid medium supplemented with 0.062 µM (wild-type and *Δzrt1zrt2* strains), 2.5 or 20 µM (all strains), and 100, 250 or 500 µM (wild-type and *Δzrc1cot1* strains) ZnSO_4_. Results are representative of three independent experiments (a replicate is shown in Figure S2C).

The transport properties of the plant ZIF2 carrier were then explored by means of heterologous expression in *Saccharomyces cerevisiae*. Correct expression of the GFP-ZIF2 fusion protein in yeast was confirmed by immunoblotting (Figure S2A) and fluorescence microscopy (Figure S2B). To determine whether the plant transporter is able to influence Zn delivery to yeast cells, the GFP-ZIF2 fusion was introduced into the *Δzrt1zrt2* and *Δzrc1cot1* mutant strains, which are sensitive to low and high Zn concentrations, respectively [31], [32]. As seen in Figures 5 and S2C, yeast cells expressing GFP-ZIF2 displayed no significant growth difference with respect to cells transformed with the empty vector in normal nutrition medium (2.5 µM Zn^2+^). Due to the lack of the Zn importers Zrt1 and Zrt2, the *Δzrt1zrt2* mutant exhibited as expected [32] a severe growth retardation under Zn-limiting conditions that was further exacerbated by GFP-ZIF2 expression. Conversely, GFP-ZIF2 was able to markedly alleviate the growth defects induced at high Zn concentrations by loss of the vacuolar transporters Zrc1 and Cot1 [31]. Similar although less pronounced trends were observed when ZIF2 was expressed in the wild-type background (Figure 5 and Figure S2C). To correlate these effects with an enhanced Zn release capacity of yeast cells, we measured the total Zn concentration of the different strains grown under various Zn conditions (Table 1). While intracellular Zn accumulation was similar for all cell types under normal Zn supply, expression of the plant carrier significantly reduced the intracellular Zn concentration in all tested backgrounds when compared with cells carrying the empty vector under both low and high Zn supplies. These findings further confirm that the ZIF2 transporter is able to mediate Zn extrusion from yeast cells.

**Table 1:** Effect of GFP-ZIF2 expression on the zinc content of yeast cells.

Genotype	External Zn^2+^ concentration (µM)		
	0.062	2.5	500
WT+*pGREG576*	2.38±0.36	2.78±0.18	118.06±17.82
WT+GFP-ZIF2	0.48±0.47 (7.23e^−4^)	2.66±0.06	41.88±27.05 (3.27e^−3^)
*Δzrt1zrt2*+*pGREG576*	1.25±0.34	2.53±0.12	——
*Δzrt1zrt2*+GFP-ZIF2	*ND*	2.52±0.16	——
*Δzrc1cot1*+*pGREG576*	——	2.81±0.02	146.78±15.56
*Δzrc1cot1*+GFP-ZIF2	——	2.81±0.03	26.89±1.26 (5.58e^−6^)

Total Zn concentration (pg/cell) of wild-type, *Δzrt1zrt2* and *Δzrc1cot1* mutant yeast cells, harbouring either the cloning vector *pGREG576* or the *pGREG576_ZIF2* plasmid, grown in Zn-free liquid medium supplemented with 0.062, 2.5 or 500 µM ZnSO_4_ (means ± SD, *n* = 4). Statistically significant differences from the respective empty-vector control are indicated with the *P* values (in parentheses) obtained by Student's *t*-test. *ND*, not detectable.

By contrast, expression of the plant transporter was unable to rescue the growth defects induced at high Co concentrations by loss of the Zrc1 and Cot1 transporters [31], nor was it able to influence yeast response to several other plant beneficial ions (Figure S3A). ZIF2, also known as ERD6 (Early Response to Dehydration)-like 7, belongs to the Monosaccharide-like Transporter subfamily of MFS transporters and indeed one of its closest *S. cerevisae* homologues is the inositol transporter Itr1 [33]. Nevertheless, GFP-ZIF2 was unable to rescue the *Δitr1* deletion mutant's deficient growth under limiting *myo*-inositol concentrations (Figure S3B). Conversely, expression of GFP-ZIF2 conferred enhanced yeast resistance to acetate, malate and citrate (Figure S3C), indicating that besides Zn, ZIF2 is also able to modulate weak acid sensitivity in yeast.

### Loss of *ZIF2* Function Causes Zn Hypersensitivity in *Arabidopsis*

To uncover the *in vivo* role of the ZIF2 transporter, we isolated an *A. thaliana* mutant allele (SALK_037804), harbouring a T-DNA insertion in the tenth intron of the *ZIF2* gene, which we named *zif2-1* (see Figure 2A). RT-PCR analysis of *ZIF2* expression in *zif2-1* homozygous seedlings using primers annealing upstream of the insertion site revealed transcript levels comparable to wild-type plants, but no expression was detected when primers flanking or annealing downstream of the T-DNA segment were used (Figure S4). This indicated that the mutant allele produces a truncated version of the *ZIF2* transcripts that lacks nearly the entire sequence corresponding to the second transmembrane domain and is thus unlikely to encode a functional membrane transporter [34], strongly suggesting that *zif2-1* is a true loss-of-function mutant.

**Figure 6:**
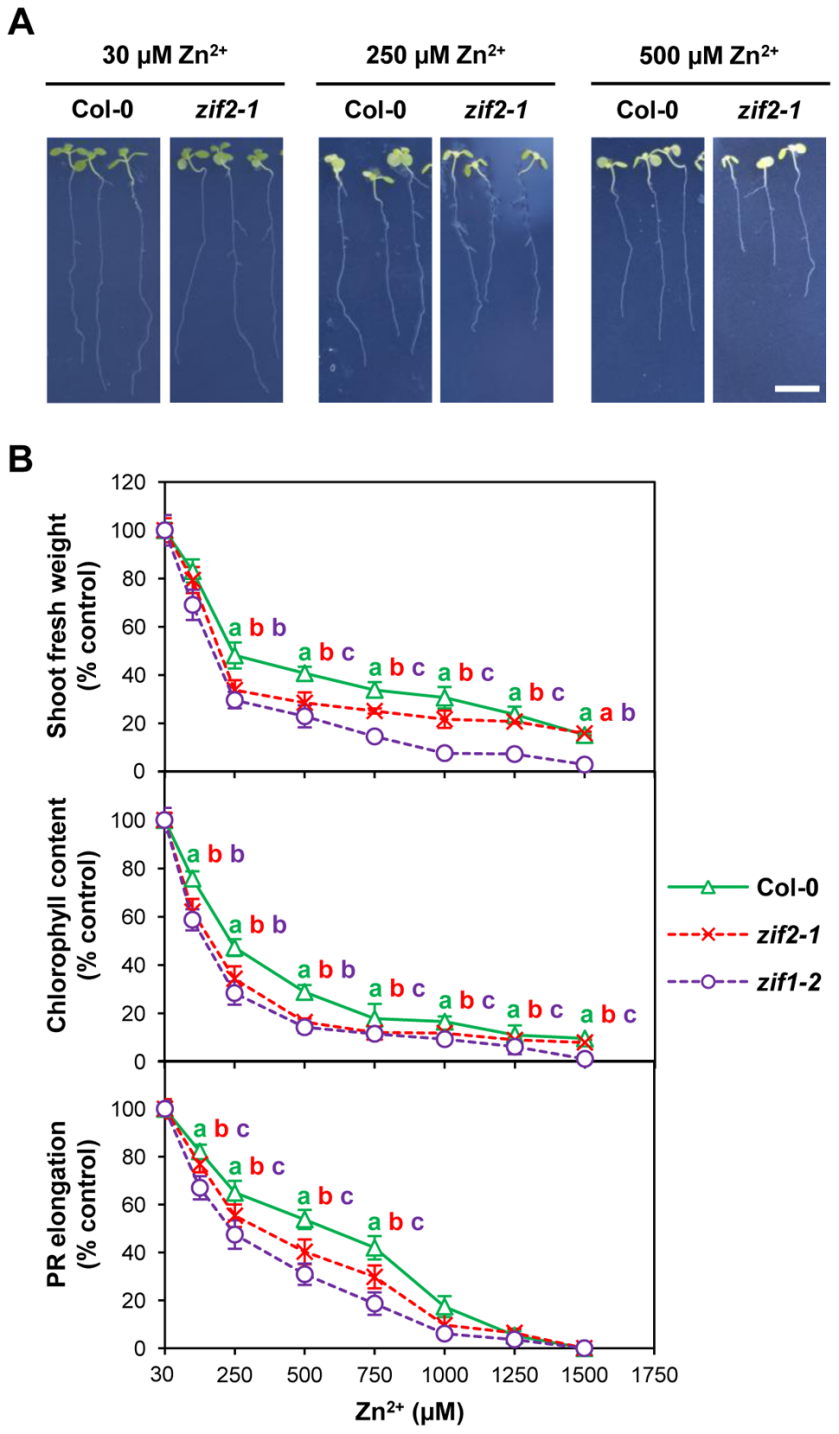
An *Arabidopsis ZIF2* loss-of-function mutant is hypersensitive to Zn toxicity. (A) Representative images of 10-d old wild-type (Col-0) and mutant (*zif2-1*) seedlings grown on control medium (30 µM Zn^2+^) or under excess Zn supply (250 and 500 µM Zn^2+^). Scale bar, 1 cm. (B) Effect of Zn toxicity on shoot biomass (upper panel), chlorophyll content (middle panel) and PR elongation (lower panel) of wild-type (Col-0) and *zif2-1* or *zif1-2* mutant seedlings. Results are representative of three independent experiments and values represent means ± SD (*n* = 8 for shoot biomass/chlorophyll content and *n* = 16 for PR elongation). Different letters indicate statistically significant differences between genotypes (*P*<0.05; Student's *t*-test).

When grown *in vitro* under optimal conditions, *zif2-1* mutant seedlings appeared morphologically indistinguishable from the corresponding wild type (Col-0), showing normal shoot growth, chlorophyll content and root system development (Figure 6A, Table S1). In the presence of excessive Zn amounts, *Arabidopsis* seedlings develop toxicity symptoms, typically including shoot growth retardation, leaf chlorosis and inhibition of PR elongation [1]. These three Zn toxicity hallmarks were visibly exacerbated in *zif2-1* when compared to the wild type following exposure to a wide Zn toxicity range, though to a lesser extent than in the previously characterised [20] z*if1-2* mutant (Figure 6). By contrast, no significant differences between *zif2-1* and wild-type seedlings could be observed at the PR elongation level under conditions of Zn deficiency, even when assayed on media with low cation content [6] (Figure S5A). In contrast to the *zif1-2* mutant [7], [20], no *zif2-1* phenotype was observed under conditions of Fe depletion (Figure S5B) or in the presence of excessive amounts of Cd or Ni (Figure S6). At least at the tested concentrations, sensitivity of the mutant to excess of other essential, beneficial or rhizotoxic cations (Figure S6) or to *myo*-inositol (Figure S7) was also unaltered, as was sensitivity to sucrose and glucose, whose protective effect on Zn toxicity was unchanged by the *zif2-1* mutation (Figure S8). Taken together, these data suggest that ZIF2 acts specifically as a Zn detoxifier in *Arabidopsis*.

To exclude the possibility that the observed phenotype results from disruption of another gene, we transformed the *zif2-1* mutant with a genomic fragment spanning the entire *ZIF2* gene and including the same promoter sequence used in the reporter gene experiments. These complementation lines exhibited complete restoration of wild-type sensitivity to excess Zn (Figure S9), thus confirming that the ZIF2 transporter participates in basal Zn tolerance in *Arabidopsis*.

### Overexpression of the *ZIF2.2* Splice Variant with Longer 5′UTR Confers Higher Plant Zn Tolerance

**Figure 7:**
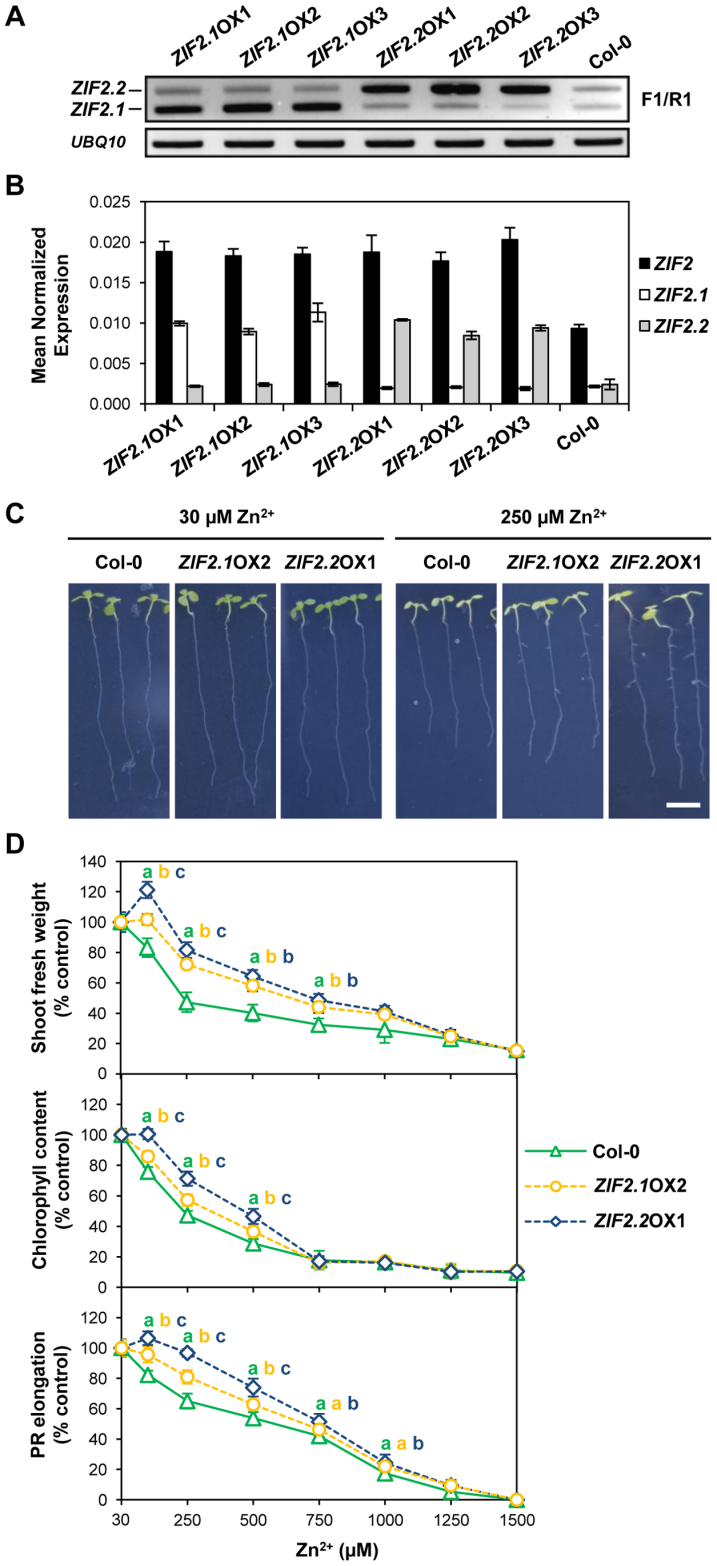
The *ZIF2.1* and *ZIF2.2* splice variants contribute differentially to Zn tolerance in *Arabidopsis*. (A) RT-PCR profile of *ZIF2.1* and *ZIF2.2* expression in roots of 7-d old seedlings of the wild type (Col-0) and *ZIF2.1* or *ZIF2.2* overexpression lines. The location of the F1 and R1 primers used is shown in Figure 2A. Expression of the *UBQ10* gene was used as a loading control. Results are representative of three independent experiments. (B) Real-time RT-PCR analysis of total *ZIF2* expression as well as of *ZIF2.1* and *ZIF2.2* transcript levels in roots of 7-d old seedlings of the wild type (Col-0) and *ZIF2.1* or *ZIF2.2* overexpression lines using *UBQ10* as a reference gene. Results are from two independent experiments and values represent means ± SE (*n* = 4). (C) Representative images of 10-d old wild-type (Col-0) and *ZIF2.1*- or *ZIF2.2*-overexpressing (*ZIF2.1*OX2 or *ZIF2.2*OX1) seedlings grown on control medium (30 µM Zn^2+^) or under excess Zn supply (250 µM Zn^2+^). Scale bar, 1 cm. (D) Effect of Zn toxicity on shoot biomass (upper panel), chlorophyll content (middle panel) and PR elongation (lower panel) of wild-type (Col-0) and *ZIF2.1*- or *ZIF2.2*-overexpressing (*ZIF2.1*OX2 or *ZIF2.2*OX1) seedlings. Results are representative of three independent experiments and values represent means ± SD (*n* = 8 for shoot biomass/chlorophyll content and *n* = 16 for PR elongation). Different letters indicate statistically significant differences between genotypes (*P*<0.05; Student's *t*-test).

To gain insight into the physiological relevance of the *ZIF2* 5′UTR intron retention event promoted in roots by high Zn exposure, we selected three transgenic *Arabidopsis* lines independently expressing either the *ZIF2.1* or *ZIF2.2* cDNA under the control of the 35S promoter in the wild-type background that similarly displayed an approximately five-fold increase in expression of either splice variant when compared to wild-type plants (Figure 7A, 7B). Importantly, our RT-PCR analyses also showed that *ZIF2.1* levels in the *ZIF2.2*-overexpressing lines were similar to those detected in the wild type, indicating that the 5′UTR intron is not spliced from the transgenic *ZIF2.2* cDNA. No obvious phenotypical alterations were observed between wild-type and *ZIF2*-overexpressing seedlings grown under control Zn conditions (Figure 7C, Table S1). However, *ZIF2.1* or *ZIF2.2* overexpression substantially attenuated the detrimental effects induced by excessive Zn amounts over a broad range of Zn supplies (Figure 7C, D; Figure S10). Together with the above results, these data demonstrate that *ZIF2* deletion and overexpression confer exact opposite phenotypes in *Arabidopsis* upon challenge with Zn excess. Interestingly, the three *ZIF2.2*-overexpressing lines consistently exhibited significantly greater Zn tolerance levels than the three overexpressing *ZIF2.1* (Figure 7C, D; Figure S10). Thus, in addition to being prevalently expressed under Zn stress (see Figure 3C, D; Figure S1), the *ZIF2.2* splice variant contributes more significantly to plant Zn tolerance than *ZIF2.1*.

### ZIF2 Function Influences Zn Tissue Partitioning in *Arabidopsis*

**Figure 8:**
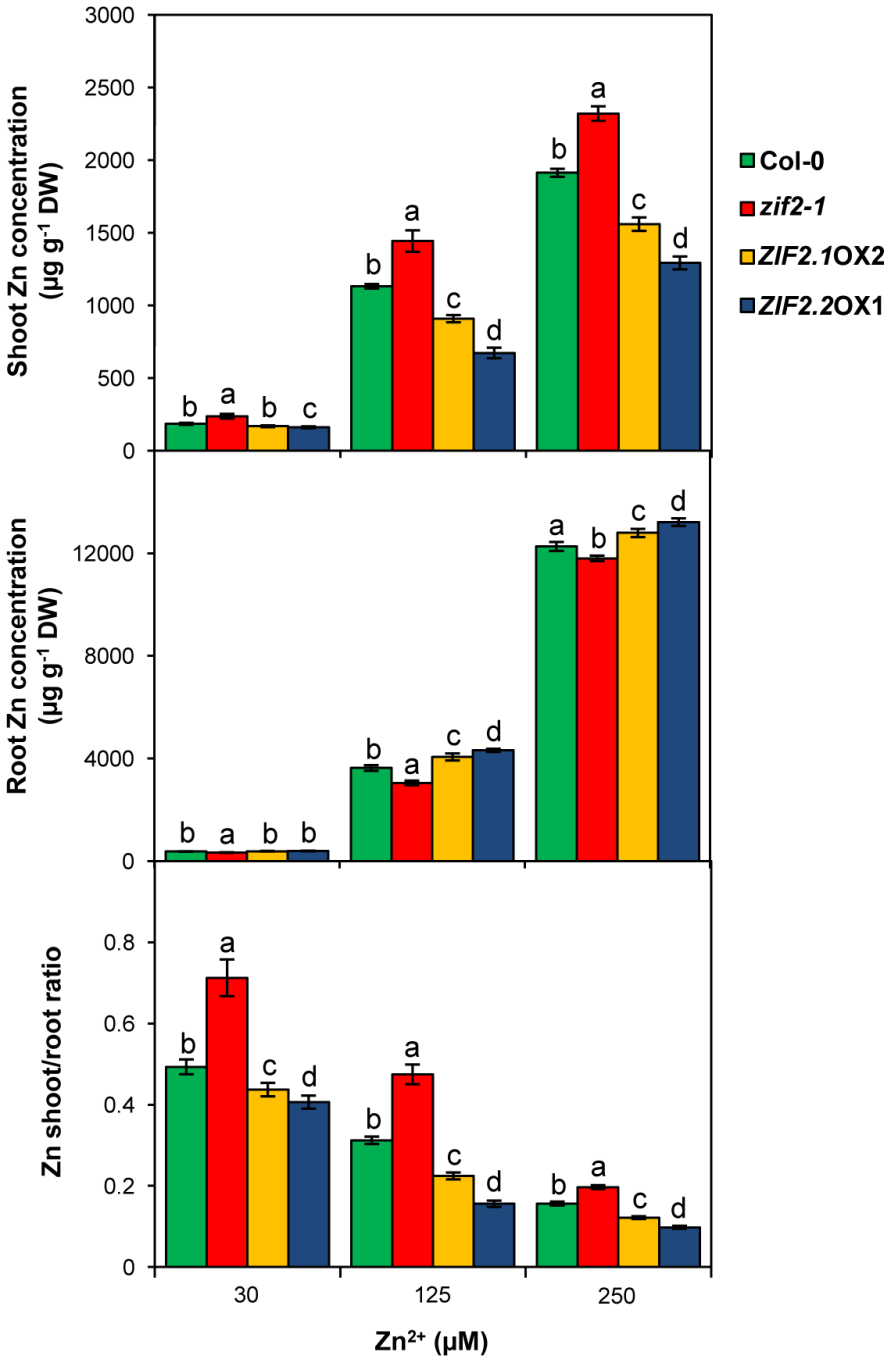
The ZIF2 transporter affects root-shoot partitioning in *Arabidopsis*. Zn concentration, expressed on a dry weight (DW) basis, in the shoot (upper panel) and root (middle panel), as well as the corresponding shoot/root ratio (lower panel), of 21-d old seedlings of the wild type (Col-0), the *zif2-1* mutant and *ZIF2*-overexpressing lines (*ZIF2.1*OX2 and *ZIF2.2*OX1) grown on control medium (30 µM Zn^2+^) or under excess Zn supply (125 and 250 µM Zn^2+^). Bars represent means ± SD (*n* = 4). Different letters indicate statistically significant differences between genotypes under each condition (*P*<0.05; Student's *t*-test).

As a first step towards unravelling the mechanisms by which the ZIF2 transporter mediates Zn tolerance in *Arabidopsis*, we determined the Zn concentration of shoot and root tissues in seedlings grown under control Zn supply or moderate Zn stress (Figure 8). As expected, the Zn concentration of both tissues increased while the corresponding Zn shoot-to-root ratio decreased commensurately with Zn supply increment. Under any of the Zn supplies tested, the above-ground parts of the *zif2-1* mutant accumulated on average 20% more Zn than wild-type shoots, whereas *zif2-1* mutant roots concentrated significantly less Zn than those of the wild type. The opposite Zn partitioning trend was observed in *ZIF2*-overexpressing plants, which accumulate respectively less and more Zn in their shoot and root tissues than wild-type plants. This tendency, while also observed under control conditions, was substantially more pronounced at the two highest Zn concentrations (Figure 8). As the total amount of Zn quantified at the whole seedling level was globally comparable in all genotypes (Figure S11), the Zn shoot-to-root ratio was enhanced and reduced by *ZIF2* loss of function and overexpression, respectively. These results indicate that ZIF2 function alters Zn partitioning between roots and shoots by driving root Zn immobilization and therefore shoot Zn exclusion, particularly under conditions of Zn excess. Importantly, the effects of *ZIF2* overexpression on Zn distribution were significantly more pronounced in plants ectopically expressing the *ZIF2* transcript with the longer 5′UTR, *ZIF2.2*, than in the *ZIF2.1* transgenics (Figure 8), in clear agreement with the physiological data obtained for both types of lines (see Figures 7 and S10).

### The *ZIF2.2* Splice Variant Produces More ZIF2 Protein *In Planta* than *ZIF2.1*

We next sought to understand the different functional impact of the two *ZIF2* splice variants on Zn tolerance and whole-plant partitioning in *Arabidopsis*. Given that *ZIF2.1*- and *ZIF2.2*-overexpressing seedlings displayed very similar steady-state levels of the respective *ZIF2* transcripts (see Figure 7A, 7B), which encode the same transporter (see Figure 2B), the differences in phenotype magnitude are unlikely to result from distinct mRNA levels and/or protein stability/activity, thus pointing to a translational regulatory mechanism.

**Figure 9:**
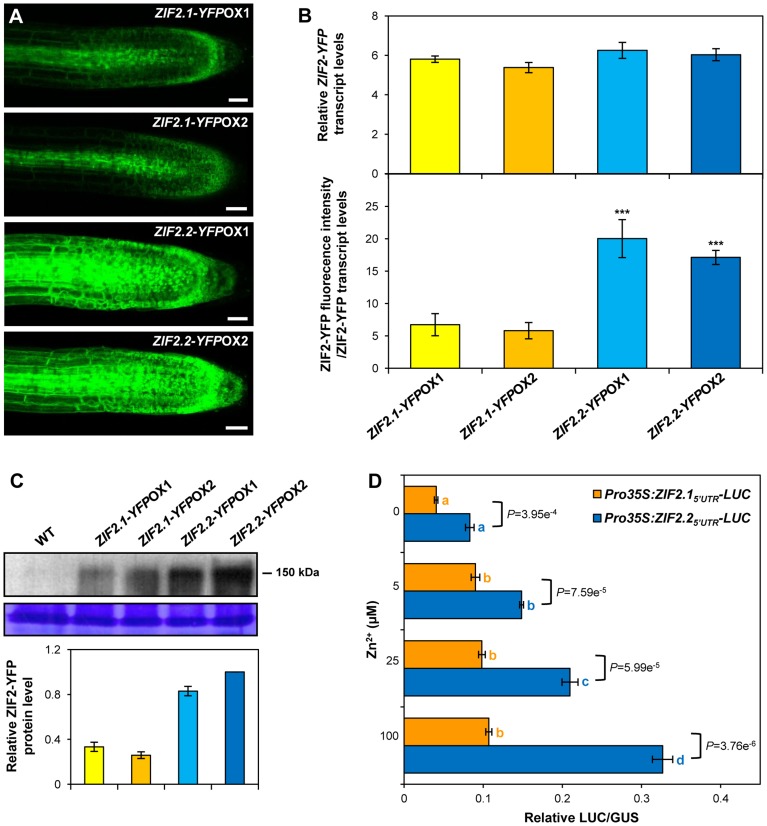
The *ZIF2.1* and *ZIF2.2* 5′UTRs exert differential effects on translation. (A) Representative confocal laser scanning microscopy images of root tips of transgenic *Arabidopsis* 7d-old seedlings expressing either the ZIF2.1-YFP or ZIF2.2-YFP fusions under the control of the 35S promoter. Detection settings for YFP visualization were identical for all genotypes. The YFP signal is visualized by green coloration. Scale bars, 50 µm. (B) Transgene transcript and protein levels in *Arabidopsis ZIF2.1-YFP* and *ZIF2.2-YFP* overexpression lines. Quantification of *ZIF2-YFP* transcript (upper panel) and corresponding fusion protein (lower panel) levels in roots of 7-d old *ZIF2-YFP* overexpression seedlings grown under control conditions. Relative *ZIF2-YFP* gene expression was quantified by real-time RT-PCR using *UBQ10* as a reference gene (means ± SD, *n* = 4). The fluorescence intensity of the YFP signal in root tips (means ± SD, *n* = 12) was normalized to *ZIF2-YFP* transcript levels to determine the relative abundance of the ZIF2-YFP protein. Asterisks denote statistically significant differences from the *ZIF2.1-YFP*OX1 line (***P<0.001; Student's *t*-test). (C) Quantification of ZIF2-YFP protein levels in *Arabidopsis ZIF2.1-YFP* and *ZIF2.2-YFP* overexpression lines. Western blot analysis of ZIF2-YFP protein levels in 5-d old seedlings from the wild type (Col-0) and *ZIF2.1-YFP*OX or *ZIF2.2-YFP*OX transgenic lines. The blot image is representative of two independent experiments and Coomassie staining is shown as a loading control. Bands were quantified and relative ZIF2-YFP protein levels plotted using the Coomassie control as a reference. The relative ZIF2-YFP protein level in the *ZIF2.2-YFP*OX1 line was set to 1. Results are from two independent experiments and values represent means ± SD (*n* = 4). (D) Effect of the *ZIF2.1* 5′UTR and *ZIF2.2* 5′UTR on translation of the *LUC* reporter gene. Transient expression of the *Pro35S:ZIF2.1_5′UTR_-LUC* or *Pro35:ZIF2.2_5′UTR_-LUC* constructs in isolated *Arabidopsis* protoplasts under various Zn supplies, using *Pro35S:GUS* as a transfection control. Results are representative of three independent experiments and values represent means ± SD (*n* = 4). Different letters indicate statistically significant differences (*P*<0.01) between Zn concentrations for each construct and *P* values for the comparison of the two constructs under each condition are shown (Student's *t*-test).

To assess the ZIF2 protein levels produced by each *ZIF2* splice variant *in planta*, we took advantage of our *ZIF2-YFP* overexpression lines (see Figure 4E, F), providing a setting in which the abundance of the ZIF2 transporter can be accurately linked to the amount of each splice variant through quantification of the corresponding YFP signal and transgene levels. Two transgenic *Arabidopsis* lines independently expressing either *ZIF2.1-YFP* or *ZIF2.2-YFP* under the control of the 35S promoter in the wild-type background were selected. Both the *ZIF2.1-YFP* and the *ZIF2.2-YFP* transgenic lines exhibited increased resistance to excess Zn when compared to the wild type (Figure S12), demonstrating the functionality of the ZIF2-YFP fusion proteins. Moreover, as with our other set of overexpression plants (see Figures 7 and S10), both *ZIF2.2-YFP* transgenic lines were significantly more tolerant to Zn toxicity than the two lines expressing *ZIF2.1-YFP* (Figure S12). Noticeably, striking differences in PR and PR tip ZIF2-YFP fluorescence levels were observed between the two types of transgenic plants, with a substantially higher ZIF2-YFP signal being detected in seedlings overexpressing the *ZIF2.2-YFP* fusion under control conditions (Figure 9A, Figure S13). Importantly, all four transgenic lines displayed comparable *ZIF2-YFP* transcript levels, with normalization of the YFP fluorescence intensity to the corresponding transgene transcript levels showing that the relative abundance of the ZIF2-YFP protein at the tonoplast of root tip cells is 2–3 fold higher in plants expressing the *ZIF2.2* splice variant (Figure 9B). Accordingly, higher ZIF2-YFP protein fusion levels were detected by western blot analysis in *ZIF2.2-YFP* than in *ZIF2.1-YFP* transgenic lines (Figure 9C). This indicated that *in planta* the *ZIF2.2* splice variant is more efficiently translated than *ZIF2.1*.

### The ZIF2 5′UTR Intron Determines Translation Efficiency in a Zn-Responsive Manner

To investigate whether the *ZIF2.1* and *ZIF2.2* 5′UTRs are sufficient to determine different translation efficiencies, the effect of each splice variant's 5′UTR on the expression of a reporter gene (*LUC*) was examined by means of an *in vivo* assay using isolated *Arabidopsis* protoplasts [35], [36]. The *ZIF2.1_5′UTR_-LUC* and *ZIF2.2_5′UTR_-LUC* constructs were expressed under the control of the 35S promoter, and LUC activity was normalized to the GUS activity of the co-transfected *Pro35S*:*GUS* reporter construct. Strikingly, and despite the fact that nearly two thirds of the *ZIF2.2-LUC* transcript was spliced into the *ZIF2.1-LUC* transcript (Figure S14A), under normal conditions (0 µM Zn) about twice the LUC activity was detected upon transfection with *ZIF2.2_5′UTR_-LUC* when compared with *ZIF2.1_5′UTR_-LUC* (Figure 9D). These results are consistent with the *in planta* data and indicate that the differences in translation efficiency of the two *ZIF2* splice variants are solely attributable to their 5′UTRs.

We next assessed the effect of Zn on translation driven by the two *ZIF2* 5′UTRs using the exact same experimental settings but in the presence of a range of Zn supplies (Figure 9D). Importantly, both constructs were expressed at equivalent levels under all conditions and none of the applied Zn challenges promoted the intron retention event in the *ZIF2.2-LUC* transcript (Figure S14A). Upon *ZIF2.1_5′UTR_-LUC* transfection, LUC activity was induced by about two-fold in the presence of 5 µM Zn but this up-regulation was not further amplified at higher Zn concentrations. However, translation of the *ZIF2.2_5′UTR_-LUC* transcript was strongly induced by Zn in a concentration-dependent manner, with a respective 2-, 2.5- and 4-fold induction in presence of 5, 25 and 100 µM Zn when compared to the absence of the metal ion (Figure 9D). By contrast, translation of the control *LUC* transcript was not significantly affected by the Zn challenge (Figure S14B). Hence, our results show that the retained intron in the *ZIF2* 5′UTR enhances translation in a Zn-responsive fashion, markedly promoting protein expression under excess Zn levels.

**Figure 10:**
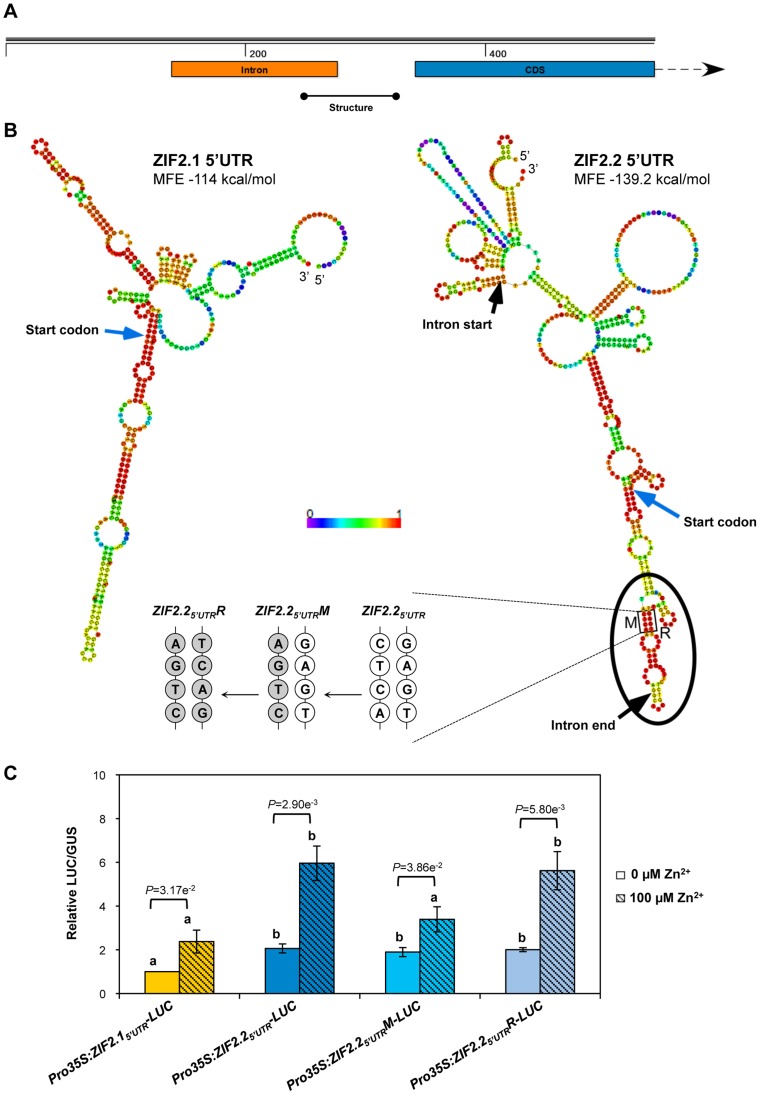
An RNA secondary structure element sustains Zn regulation of translation driven by the *ZIF2.2* 5′UTR. (A) Organization of the 5′ end of the *ZIF2* gene. The orange and blue rectangles represent the intronic and coding sequences, respectively, and the region forming a stable RNA structure is indicated as a black line. (B) Secondary structure predictions for the *ZIF2.1* and *ZIF2.2* 5′UTRs flanked by 200 bp of the coding sequence. A potentially stable secondary structure element is enclosed in an oval. Four consecutive bases in the *ZIF2.2* 5′UTR (*ZIF2.2_5′UTR_*) were mutated (*ZIF2.2_5′UTR_M*) by site-directed mutagenesis to destabilize the structure, which was then restored by four complementary mutations (*ZIF2.2_5′UTR_R*). Mutated bases are shown in grey. The two black arrows indicate the start and end of the intron while the blue arrows point to the start codon. The colour code indicates base pairing probabilities (red – high, blue – low) and the minimum free energy (MFE) of the entire 5′UTR (+ 200 nt of coding sequence) structures is indicated. (C) Transient expression of the *Pro35S:ZIF2.1_5′UTR_-LUC*, *Pro35S:ZIF2.2_5′UTR_-LUC*, *Pro35S:ZIF2.2_5′UTR_M-LUC* (destabilized structure element) or *Pro35S:ZIF2.2_5′UTR_R-LUC* (restored structure element) constructs in isolated *Arabidopsis* protoplasts under 0 or 100 µM Zn supplies. *Pro35S:GUS* was used as a transfection control and relative LUC/GUS activity for *Pro35S:ZIF2.1_5′UTR_-LUC* under 0 µM Zn was set to 1. Results are from four biological repetitions and values represent means ± SD (*n* = 4). Different letters indicate statistically significant differences (*P*<0.05) between the different constructs for each Zn concentration, and *P* values for the comparison of the two Zn concentrations for each construct are shown (Student's *t*-test).

Interestingly, secondary structure prediction of the *ZIF2.2* 5′UTR indicated the formation of a stable imperfect stem loop immediately upstream of the ATG codon (Figure 10A, B), which is predicted regardless of the length of *ZIF2* sequence used, even with the full-length transcript. This structure is lost in the *ZIF2.1* splice variant as the intron removes roughly half of the sequence participating in the structure formation. To determine whether the predicted 5′UTR secondary structure element plays a role in translational regulation of the two alternatively-spliced mRNAs, we performed two successive rounds of site-directed mutagenesis on the *ZIF2.2_5′UTR_-LUC* construct to generate the *ZIF2.2_5′UTR_M-LUC* (where CTCA was mutated to AGTC) and *ZIF2.2_5′UTR_R-LUC* (where TGAG was mutated to GACT) constructs, in which the secondary structure was destabilized and restored, respectively (Figure 10B). In the absence of Zn, a two-fold increase in LUC activity was detected upon transfection with any of the three *ZIF2.2_5′UTR_-LUC* constructs when compared with the *ZIF2.1_5′UTR_-LUC* transcript (Figure 10C). However, destabilization of the secondary structure strikingly reduced the pronounced inductive effect that Zn exerts on *ZIF2.2_5′UTR_-LUC* translation, while restoration of the structure fully rescued translational induction by the metal ion (Figure 10C). The results from this last experiment indicate that the mechanism(s) underlying Zn regulation of translation driven by the *ZIF2* 5′UTR depend, at least to a large extent, on an RNA secondary structure that is present when the 5′UTR intron is retained.

## Discussion

Since the identification of ZIPs as the first plant Zn transporters [37], several carriers from well-established metal transporter families have been linked to plant Zn homeostasis. The few plant MFS carriers characterised so far have been mainly implicated in sugar, nitrate, oligopeptide and phosphate transport [38]–[40]. Here we report that besides ZIF1 [20], another MFS carrier contributes to *Arabidopsis* Zn tolerance, hinting at a broader role for this class of transporters in plant heavy-metal homeostasis.

Conclusive evidence for a ZIF2 role in basal Zn tolerance stems from our functional analysis of *Arabidopsis ZIF2* loss-of-function and overexpression lines, exhibiting respectively enhanced sensitivity and resistance to Zn toxicity. The ZIF2 tonoplastic localisation detected *in planta* is consistent with two previous proteomic studies of the *Arabidopsis* vacuole [41], [42] and strongly suggests that ZIF2 is involved in vacuolar Zn compartmentalisation. Indeed, plant vacuoles play an essential role during metal detoxification and sequester up to 80% of cellular Zn [43]. In agreement with our physiological data, Zn concentration determination in seedlings exposed to high Zn shows that the shoot-to-root ratio of the *zif2-1* mutant is unwedged to the shoot, while Zn is preferentially retained in *ZIF2*-overexpressing roots, demonstrating that ZIF2 function influences Zn root-to-shoot translocation under Zn excess. Given that *ZIF2* is primarily expressed in the root endodermis and cortex, we propose that the encoded transporter protects from Zn toxicity by promoting vacuolar immobilization of the metal ion in these two cell layers, thus restricting symplastic movement towards the stele and subsequent xylem loading and translocation to the shoot. Richard et al. [44] showed that low root-driven Zn translocation rates to the shoot contribute to higher Zn tolerance, as reported for the *Arabidopsis* HMA3, MTP3, MTP1 and ZIF1 [10], [19], [20], [45]. The partial expression pattern overlap among these vacuolar transporters and ZIF2 suggests either some degree of functional redundancy or a concerted action to regulate root Zn symplastic movement. Putative roles of the ZIF2 transporter in other plant organs where substantial *ZIF2* expression was detected, such as in flowers, cannot be excluded as already shown for instance for the HMA2 and HMA4 transporters. Similarly to *ZIF2*, *HMA2* and *HMA4* promoter activity is high in developing anthers, and a double *hma2hma4* mutant exhibits a male-sterile phenotype [17].

The identification of a transporter's physiological substrate(s) can be instrumental in elucidating the molecular mechanisms governing its function, but remains an extremely challenging task. Importantly, our yeast heterologous expression and *in planta* phenotypical data point to a ZIF2 effect restricted to Zn, similarly to MTP1 and MTP3 that exhibit relatively high Zn selectivity [10], [45] but in contrast to HMAs or ZIPs that often display broad metal selectivity contributing to plant metal homeostasis crosstalk [19], [46]. More puzzling are our preliminary findings indicating that, despite belonging to the Monosaccharide-like subfamily, activity of the ZIF2 transporter does not seem to influence sugar sensitivity *in planta* nor in yeast. Instead, heterologous expression studies show that ZIF2 is able to mediate Zn efflux in yeast cells without requiring additional plant-specific factors, as already reported for HMA2, HMA4, MTP1 and PCR2 [5], [11], [16]. By contrast, the transport activity of ZIF1 is unable to complement a Zn hypersensitive yeast mutant, thus indicating that the mode of action of the two MFS carriers in mediating Zn tolerance is strikingly different. Haydon et al. [7] identified the substrate of ZIF1 as nicotianamine, a low molecular mass chelator with high affinity for a range of transition metals, and proposed that ZIF1, by affecting nicotianamine vacuolar partitioning, specifically promotes Zn vacuolar sequestration via Zn^2+^/H^+^ antiporters. As no plasma-membrane Zn exporter has been identified so far in *S. cerevisiae* [47], it seems reasonable to exclude ZIF2 activation of endogenous transporters catalysing Zn efflux. On the other hand, as MFS transporters are known to transport small solutes in response to chemiosmotic gradients and as ZIF2 affects weak acid delivery in yeast, it is tempting to speculate that ZIF2 indirectly mediates Zn vacuolar sequestration by transporting a small chelator or organic acid or by influencing the tonoplastic proton gradient. Indeed, plant cells are known to take advantage of proton gradients to actively sequester ions inside the vacuole [48] and the *Arabidopsis* H^+^-ATPase (V-ATPase), which energizes transport across the tonoplast, is required for full Zn tolerance [49].

Our results also indicate that the Zn metal ion positively regulates *ZIF2* expression at multiple levels. Firstly, root *ZIF2* transcript levels are substantially induced following exposure to elevated Zn supplies, corroborating an essential role in Zn detoxification, as already inferred for ZIF1 [20] or MTP3 [10]. Secondly, splicing of the *ZIF2* pre-mRNA is Zn-regulated, with high levels of the metal favouring retention of the 5′UTR intron and hence expression of the longer *ZIF2.2* splice variant. The splicing factors regulating this intron retention event remain unknown. In animal systems, splice site selection is known to be primarily regulated by the highly conserved serine/arginine-rich (SR) and heterogeneous nuclear ribonucleoprotein particle (hnRNP) protein families, which bind specific exonic or intronic sequences in the pre-mRNA. While these splicing factors have been implicated in the modulation of alternative splicing also in plants, their direct endogenous targets and the mechanisms controlling differential splice site usage have not been elucidated [50], [51].

Noticeably, our data indicate that the 5′UTR intron is not spliced from the *ZIF2.2* transgene in our *ZIF2.2*-overexpressing lines, while splicing of this intron occurs at considerable rates from the *ZIF2.2_5′UTR_-LUC* transcript transiently expressed in protoplasts. On the other hand, the inductive effect that Zn exerts on the intron retention event in wild-type roots is lost in the single-cell system. These apparent discrepancies could be due to the distinct tissue sources (roots versus leaves) and/or to differences in transcription rates, which are well known to affect alternative splicing [52], between the different expression contexts. The molecular mechanisms underlying the regulation of alternative splicing in plants are poorly understood. Despite numerous reports that abiotic stress markedly affects plant mRNA splicing, virtually nothing is known about the effects of metal ions. Interestingly, in humans Zn has been shown to modulate alternative splicing of the hypoxia-inducible factor *HIF1* [53] and the pro-apoptotic *Bim* [54] genes.

Finally, and given that information on the functional relevance of alternative splicing in plant systems is also surprisingly scarce, a key finding of this study is that two *ZIF2* splice forms differentially contribute to Zn detoxification. In fact, our two sets of overexpression data indicate not only that *ZIF2.2* confers greater Zn tolerance than *ZIF2.1*, but also that the different functional impact of the two mRNAs is not due to differences in their steady state levels. As the two alternative transcripts encode the same transporter, it is also highly improbable that differences in protein stability or activity account for the observed variations in resistance to Zn toxicity. Instead, we show that the stronger Zn tolerance phenotype conferred by *ZIF2.2* results from its enhanced translation efficiency. Interestingly, Zn significantly promotes translation of both *ZIF2* splice variants, but this effect is dramatically more pronounced for *ZIF2.2*. Furthermore, our results indicate that the *ZIF2* 5′UTR alone is sufficient for this translational control. Indeed, 5′UTR sequence and structural features have been proposed to play an essential role in translational regulation in plants [55], [56], as described for other eukaryotes. It is interesting to note that expression of an *Arabidopsis* tonoplastic Zn^2+^/Mg^2+^ transporter, AtMHX, was shown to be repressed at the translational level through the inhibitory effect of an upstream open-reading-frame present in the 5′UTR of the corresponding gene [57], [58]. Importantly, we were able to demonstrate that the mechanism(s) underlying Zn-responsive *ZIF2.2* translation depend largely on a predicted stable stem loop structure that is lost when the *ZIF2* 5′UTR intron is spliced out. From the publicly available databases, the 5′UTR intron retention event in *ZIF2* appears to be fairly specific to this *Arabidopsis* gene, as no significant sequence homology is found with other genes in *A. thaliana* nor in other plant species except with the *ZIF2* orthologue in *Arabidopsis lyrata*. Similarly, the identified stable stem loop appears to be a unique feature of *A. thaliana*, as no common mRNA secondary structure could be predicted in the 5′UTR of plant *ZIF2* orthologues.

Selective recruitment of certain physiologically relevant mRNAs under stress conditions that trigger a global repression of the initiation step of translation has been widely reported in metazoans. Such a mechanism is beginning to emerge as a key process in plant adaptation to environmental stresses, interestingly for instance during sub-lethal cadmium poisoning [59]. In particular, secondary structures in the 5′UTRs have been implicated in cap-independent mRNA translation in maize under heat and other stress conditions [60], [61] as well as in *Arabidopsis* under heat stress [55]. The findings presented here indicate that alternative splicing controls the levels of a Zn stress responsive mRNA variant of the ZIF2 transporter to enhance plant tolerance to the metal ion.

## Materials and Methods

### Plant Materials and Growth Conditions

The *Arabidopsis thaliana* (L.) Heynh., ecotype Colombia (Col-0), was used in all experiments. Seeds of the T-DNA insertion mutants *zif2-1* (SALK_037804) and *zif1-2* (SALK_011408; [20]) were obtained from the Nottingham Arabidopsis Stock Centre (NASC) (Nottingham, UK). The exact *zif2-1* T-DNA insertion site was confirmed using gene-specific primers (Table S2) and primers annealing at the T-DNA border, which also allowed PCR-based genotyping to identify homozygous lines. Plant transformation was achieved by the floral-dip method [62] using *Agrobacterium tumefaciens* strain EHA105. Seeds were surface-sterilized and sown on Murashige and Skoog [63] medium solidified with 0.8% agar, stratified for 3 d, placed in a growth chamber and transferred to soil after 2–3 weeks. Plants were cultivated under long-day conditions (16-h light, 22°C/8-h dark, 18°C; 60% RH).

### Gene Expression Analyses

RLM-5′RACE was performed using the FirstChoice RLM-RACE kit (Ambion) on total RNA extracted from 21-d old seedlings, adult leaves, flowers or roots exposed to high Zn according to the manufacturer's instructions and using various gene-specific primers (Table S2). Subsequently, PCR products were purified, cloned into the p-Gem-T-easy vector (Promega) and sequenced. For native *ZIF2* promoter reporter gene experiments, a fragment including the 1233 bp immediately upstream of the start codon was PCR-amplified (Table S2) from genomic DNA and inserted via the *Sac*I/*Sac*II restriction sites into the pKGWFS7 plasmid [64]. After agroinfiltration of this *ProZIF2:GFP:GUS* construct into wild-type plants, eight independent transformants all showing similar tissue-specific GUS expression patterns were recovered. Histochemical staining of GUS activity was performed as described by Sundaresan et al. [65]. RT-PCR analyses were conducted as previously described [66] using primers designed to detect *ZIF2*, *ZIF1*, *ZIP1*, *ROC10 (CYCLOPHILIN)* and *UBQ10 (UBIQUITIN10)* expression (Table S2). The results shown are representative of three independent experiments. Real-time RT-PCR was performed using specific primers (Table S2) on a CFX384 Touch Real-Time PCR Detection System (Bio-Rad) using the Absolute SYBR Green ROX mix (Thermoscientific) according to the manufacturer's instructions. For each condition tested, two RNA extractions from different biological samples and two reverse transcription reactions for each biological repeat were performed. Data were processed using Q-Gene [67] that took the respective primer efficiency into consideration.

### Subcellular Localisation Studies

To generate ZIF2 protein fusions with the YFP and GFP reporters, each *ZIF2* transcript (5′UTR+coding sequence except the stop codon) was PCR-amplified (Table S2) using root cDNA as a template, and independently inserted under the control of the 35S promoter via the *Xho*I/*Pac*I restriction sites into the YFP- or GFP-tagged versions of the pBA002 vector. Two transgenic lines displaying a strong fluorescence signal in the root were recovered upon transformation of wild-type plants with either *Pro35S:ZIF2.1-YFP* or *Pro35S:ZIF2.2-YFP*. These YFP constructs were also transfected into *Arabidopsis* protoplasts (see below). Transient co-expression of the GFP constructs with the tonoplast marker γ-Tonoplast Intrinsic Protein (TIP)-mCherry or the plasma membrane marker Plasma membrane Intrinsic Protein 2A (PIP2A)-mCherry [30] and the pBIN-NA construct [68] in leaf abaxial epidermal cells of *Nicotiana tabacum* was performed via the agroinfiltration procedure described by Voinnet et al. [69] using *A. tumefaciens* strain GV3101.

### Yeast Manipulations

The *S. cerevisiae* mutant strains *Δzrt1zrt2* [32] and *Δzrc1cot1* [31], along with the corresponding wild-type strain DY1457, were used in the Zn experiments. All other studies were performed in the parental strain BY4741 and the derived deletion mutant *Δitr1* [33]. Cloning of the *Arabidopsis ZIF2* full-length coding sequence into the *pGREG576* vector ([70]; Euroscarf collection) and expression analysis of the corresponding GFP-ZIF2 fusion protein by fluorescence microscopy and western blotting were performed as described previously [71]. Growth curve and spot assays were conducted in MMB-U liquid or agarized medium [71] supplemented with the indicated compound (Sigma-Aldrich) at the desired concentration. Results presented are representative of three independent experiments.

### Plant Phenotypical Assays

All assays were performed in a climate-controlled growth cabinet under long-day conditions. After 5 d of vertically-oriented growth on control medium (30 µM Zn), seedlings were transferred to fresh medium containing the indicated compound (Sigma-Aldrich) at the specified concentration. PR elongation, along with shoot biomass and chlorophyll content [72] were evaluated after an additional 1 and 3 weeks of growth, respectively. Results are representative of at least three independent experiments.

### Genomic Complementation and Overexpression Analyses

For genomic complementation, a 4450-bp fragment encompassing the entire *ZIF2* gene and including the 1233-bp promoter sequence described above was PCR-amplified (Table S2) from genomic DNA and inserted into the promoterless version of pBA002 via the *Nco*I/*Xba*I restriction sites. Representative results for three lines recovered upon introduction of the construct into *zif2-1* mutant plants are shown.

*ZIF2* overexpression constructs were generated as described for the YFP and GFP plasmids, except that the corresponding fragments were inserted into the pBA002 background via the *Xho*I/*Asc*I restriction sites. After agroinfiltration of wild-type plants, three transgenic lines independently overexpressing each *ZIF2* transcript were selected.

### Protoplast Transient Expression Assays

*Arabidopsis* protoplasts were generated as described by Yoo et al. [35] and transfected by polyethylene glycol transformation [73]. To generate the *Pro*35S:*ZIF2*_5′UTR_ fusion constructs with the *LUC* reporter, the *ZIF2.1* and *ZIF2.2* 5′UTRs were PCR-amplified (Table S2) and independently cloned into the *Pro35S:LUC* vector [35] via the *Sal*I/*Nco*I restriction sites. Site-directed mutagenesis of the *Pro35S:ZIF2.2_5′UTR_-LUC* construct was performed using gene-specific primers (Table S2) and the NZYMutagenesis kit (NZYTech) according to the manufacturer's instructions. Protoplast transient expression assays along with GUS and LUC activity measurements and RNA protoplast extraction were performed as described previously [74]. Results shown are representative of at least three independent experiments.

### Microscopy and Western Blot Analysis

Differential interference contrast and confocal images were taken with a DM LB2 microscope (Leica) and an LSM 510 laser scanning microscope equipped with a Meta detector (Zeiss), respectively. Excitation/detection wavelengths used to detect fluorescence were 488/500–550 nm for GFP, 514/535–590 nm for YFP, 543/565–615 nm for mCherry, 543/>560 nm for propidium iodide and 458/>560 nm for autofluorescence.

For YFP signal quantification, fluorescence detection parameters (laser intensity, offset, gain and pinhole settings) were set so the fluorescence signal emitted by *ZIF2.2-YFP*OX1 root tips was just below the saturation threshold. Twelve micrographs per genotype were captured in the median section of roots from 5-d old seedlings immerged in 50 mM Phosphate Buffer using identical confocal settings to allow comparison between genotypes. Post-imaging, average fluorescence intensity within the whole region (ca. 20 µm^2^) spanning the extreme root apex to the first elongating cells or the zone immediately above the first elongating cells was recorded. Results are representative of three independent experiments.

Total protein was extracted from 5-d old seedlings using 2X Laemmli Extraction Buffer [75] and equal amounts of extracts were resolved on a 8% SDS/polyacrylamide gel before proteins were transferred to a PVDF membrane (Immobilon-P, Millipore), which was incubated with anti-GFP primary antibody (Roche; 1∶500 dilution) and then with anti-mouse peroxydase-conjugated secondary antibody (Amersham Pharmacia; 1∶20000 dilution), before membrane-associated peroxydase activity was revealed by ECL.

### Zinc Measurements

To measure plant zinc concentration, pooled shoot and root tissues from 3-week old seedlings grown on 30, 125 or 250 µM Zn were processed as previously described [66]. The total Zn concentration of yeast cells was measured as described by Arrivault et al. [10]. The zinc concentration of the digests was quantified using the Atomic Emission Spectrometry – Inductively Coupled Plasma — Optical Emission System (Perkin-Elmer Optical Emission, *Optima 2100 DV*) at the Laboratório de Análises, Instituto Superior Técnico (Lisbon, Portugal) according to method 3120B described by Eaton et al. [76]. Zinc standards for analytical calibration were from Merck KGaA. Four independent samples were processed per genotype.

### Software

Real-time RT-PCR results were analysed using the CFX Manager 3.0 software (Bio-Rad) and the Q-Gene application [67]. Microscopy images and scanned images of root assays and western blots were processed using LSM 510 software (Zeiss) and ImageJ (http://rsbweb.nih.gov/ij/), respectively. The entire 5′UTR (plus 200 nt of coding sequence) alternative structures and their minimum free energies (MFEs) were calculated using RNAFold [77]. The structures presented in Figure 10 are the centroid representations calculated with default parameters.

## Supporting Information

Figure S1 Expression kinetics of the *ZIF2.1* and *ZIF2.2* splice variants under zinc toxicity. RT-PCR profile of *ZIF2.1* and *ZIF2.2* expression in roots of 7-d old wild-type (Col-0) seedlings challenged for 0, 24, 48 or 96 h with 250 µM Zn. The location of the F1 and R1 primers used is shown in Figure 2A. Expression of the *UBQ10* gene was used as a loading control. Results are representative of two independent experiments.
(PDF)

Figure S2 Heterologous expression of the *Arabidopsis* ZIF2 transporter in yeast. (A) Western blot analysis of the GFP-ZIF2 fusion protein in wild-type yeast cells harbouring either the cloning vector *pGREG576* or the GFP-ZIF2-encoding *pGREG576_ZIF2* plasmid after induction of recombinant protein production using anti-GFP antibodies. (B) Representative fluorescence microscopy images of wild-type yeast cells harbouring either the cloning vector *pGREG576* (background fluorescence) or the GFP-ZIF2-encoding *pGREG576_ZIF2* plasmid after induction of recombinant protein production, suggesting that the GFP-ZIF2 fusion protein is targeted to the yeast plasma membrane. Scale bars, 1 µm. (C) Comparison of the growth curves of non-adapted wild-type, *Δzrt1zrt2* and *Δzrc1cot1* mutant yeast cells, harbouring either the cloning vector *pGREG576* or the GFP-ZIF2-encoding *pGREG576_ZIF2* plasmid, in Zn-free liquid medium supplemented with 0.062 µM (wild-type and *Δzrt1zrt2* strains), 2.5 or 20 µM (all strains), and 100, 250 or 500 µM (wild-type and *Δzrc1cot1* strains) ZnSO_4_. Results are representative of three independent experiments (a replicate is shown in Figure 5).
(PDF)

Figure S3 Further phenotypical characterisation of yeast cells expressing the *Arabidopsis* ZIF2 transporter. (A) Comparison of the growth curves of non-adapted wild-type and Δ*zrc1cot1* mutant yeast cells, harbouring either the cloning vector *pGREG576* or the GFP-ZIF2-encoding *pGREG576_ZIF2* plasmid, in liquid medium supplemented with 3.5 mM CoSO_4_, 1 mM NiSO_4_, 1 mM CuCl_2_, 40 mM FeSO_4_ or 25 mM MnCl_2_. Results are representative of at least two independent experiments. (B–C) Susceptibility to *myo*-inositol (B) and weak acids (C) of wild-type or Δ*itr1* mutant yeast cells, harbouring either the cloning vector *pGREG576* or the GFP-ZIF2-encoding *pGREG576_ZIF2* plasmid, determined by spotting dilution series of cell suspensions (1, 1∶5, and 1∶10).
(PDF)

Figure S4 *ZIF2* expression in the *Arabidopsis zif2-1* mutant. RT-PCR analysis of *ZIF2* expression in 14-d old wild-type (Col-0) and mutant (*zif2-1*) seedlings. The location of the F1′, R1, F2, R2, F3 and R3 primers used is shown in Figure 2A. Expression of the *UBQ10* gene was used as a loading control. Results are representative of three independent experiments.
(PDF)

Figure S5 Phenotype of the *Arabidopsis zif2-1* mutant under zinc or iron deficiency. Effect of Zn deficiency under different MS strengths (A) and of ferrozine-induced Fe deficiency (B) on PR elongation of wild-type (Col-0) and *zif2-1* or *zif1-2* mutant seedlings. Results are representative of two independent experiments and values represent means ± SD (*n* = 16). Asterisks denote statistically significant differences from the wild type under each condition (***P<0.001; Student's *t*-test).
(PDF)

Figure S6 Supplemental ion response characterisation of the *Arabidopsis zif2-1* mutant. Effect of excess of essential/beneficial cations and of rhizotoxic cations on shoot biomass (upper panel), chlorophyll content (middle panel) and PR elongation (lower panel) of wild-type (Col-0) and mutant (*zif2-1*) seedlings. Results are representative of two independent experiments and values represent means ± SD (*n* = 16). No statistical differences between mutant and wild type were detected under each condition (*P*>0.05; Student's *t*-test).
(PDF)

Figure S7 Phenotypical characterisation of the *Arabidopsis zif2-1* mutant in the presence of different concentrations of *myo*-inositol. Effect of *myo*-inositol on PR elongation of wild-type (Col-0) and mutant (*zif2-1*) seedlings. Results are representative of two independent experiments and values represent means ± SD (*n* = 16). No statistical differences between mutant and wild type were detected under each condition (*P*>0.05; Student's *t*-test).
(PDF)

Figure S8 Phenotypical characterisation of the *Arabidopsis zif2-1* mutant in the presence of both zinc and sugar. Effect of sucrose or glucose combined with Zn on shoot biomass (upper panel), chlorophyll content (middle panel) and PR elongation (lower panel) of wild-type (Col-0) and mutant (*zif2-1*) seedlings. Results are representative of two independent experiments and values represent means ± SD (*n* = 8 for shoot biomass/chlorophyll content and *n* = 16 for PR elongation). Asterisks denote statistically significant differences from the wild type under each condition (**P<0.01, ***P<0.001; Student's *t*-test).
(PDF)

Figure S9 Genomic complementation of the *Arabidopsis zif2-1* mutant phenotype. Effect of Zn toxicity on shoot biomass (upper panel), chlorophyll content (middle panel) and PR elongation (lower panel) of seedlings of the wild type (Col-0), the *zif2-1* mutant and three independent genomic complementation lines, *zif2-1comp1-3*. Results are representative of three independent experiments and values represent means ± SD (*n* = 8 for shoot biomass/chlorophyll content and *n* = 16 for PR elongation). Asterisks denote statistically significant differences from the wild type under each condition (*P<0.05, **P<0.01, ***P<0.001; Student's *t*-test).
(PDF)

Figure S10 Phenotype of *Arabidopsis ZIF2.1* and *ZIF2.2* overexpression lines. Effect of Zn toxicity on shoot biomass (upper panel), chlorophyll content (middle panel) and PR elongation (lower panel) of wild-type (Col-0) and *ZIF2.1*- or *ZIF2.2*-overexpressing (*ZIF2.1*OX1-3 or *ZIF2.2*OX1-3) seedlings. Results are representative of three independent experiments and values represent means ± SD (*n* = 8 for shoot biomass/chlorophyll content and *n* = 16 for PR elongation). Different letters indicate statistically significant differences between genotypes under each condition (*P*<0.05; Student's *t*-test).
(PDF)

Figure S11 Whole-seedling zinc concentration of *Arabidopsis* wild-type, *zif2-1* mutant and *ZIF2* overexpression lines. Zn concentration, expressed on a dry weight (DW) basis, in 21-d old seedlings of the wild type (Col-0), the *zif2-1* mutant and *ZIF2*-overexpressing lines (*ZIF2.1*OX2 and *ZIF2.2*OX1) grown on control medium (30 µM Zn^2+^) or under excess Zn supply (125 or 250 µM Zn^2+^). Bars represent means ± SD (*n* = 4). No statistical differences between genotypes were detected under each condition (*P*>0.05; Student's *t*-test).
(PDF)

Figure S12 Phenotype of *Arabidopsis ZIF2.1-YFP* and *ZIF2.2-YFP* overexpression lines. Effect of Zn toxicity on PR elongation of wild type (Col-0) and *ZIF2.1-YFP* or *ZIF2.2-YFP* overexpression lines (*ZIF2.1-YFP*OX1-2 or *ZIF2.2-YFP*OX1-2) seedlings. Results are representative of two independent experiments and values represent means ± SD (*n* = 16). Different letters indicate statistically significant differences between genotypes under each condition (*P*<0.05; Student's *t*-test).
(PDF)

Figure S13 Quantification of ZIF2-YFP protein levels in *Arabidopsis ZIF2-YFP* overexpression lines. Quantification of the YFP signal by confocal laser scanning microscopy in primary root tip (upper panel) and primary root (lower panel) cells from 5-d old seedlings of the *ZIF2.1-YFP*OX and *ZIF2.2-YFP*OX transgenic lines. In each panel, detection settings for YFP visualization were identical for all genotypes. Results are representative of three (upper panel) or two (lower panel) independent experiments and average fluorescence (pixel) intensity is shown (means ± SD, *n* = 12). Asterisks denote statistically significant differences from the *ZIF2.1-YFP*OX1 line (***P<0.001; Student's t test).
(PDF)

Figure S14 Effect of zinc on transgene mRNA expression and luciferase (LUC) activity driven by the 35S promoter. (A) Real-time RT-PCR analysis of *ZIF2.1_5′UTR_-LUC* and *ZIF2.2_5′UTR_-LUC* transcript levels in protoplasts transiently expressing the *Pro35S:ZIF2.1_5′UTR_-LUC* (orange bars) or *Pro35:ZIF2.2_5′UTR_-LUC* (blue bars) constructs under various Zn supplies, using *UBQ10* as a reference gene. Results are from two independent experiments and values represent means ± SE (*n* = 4). No statistical differences were detected for each transcript between the different conditions (*P*>0.05; Student's *t*-test). (B) Protoplast transient expression assay using the *Pro35S:LUC* (reference control) and *Pro35S*:GUS_mutated_ (negative control, no detectable LUC activity) constructs under various Zn supplies. Results are representative of three independent experiments, corresponding to the same assay shown in Figure 9C. Values represent means ± SD (*n* = 4). *P* values inside the bars indicate statistical differences for the *Pro35S:LUC* construct (comparison with 0 µM Zn condition) obtained by Student's *t*-test.
(PDF)

Table S1 Growth parameters of *Arabidopsis ZIF2* loss-of-function and overexpression lines under control conditions.
(PDF)

Table S2 Sequences of primers used.
(PDF)
