# Supplementary material for: Intron Retention in the 5′UTR of the Novel ZIF2 Transporter Enhances Translation to Promote Zinc Tolerance in Arabidopsis
Source: PLoS Genet. 2014 May 15;10(5):e1004375. doi: 10.1371/journal.pgen.1004375 (PMC4022490; doi:10.1371/journal.pgen.1004375)
Supplement: Figure S1 — Expression kinetics of the ZIF2.1 and ZIF2.2 splice variants under zinc toxicity. RT-PCR profile of ZIF2.1 and ZIF2.2 expression in roots of 7-d old wild-type (Col-0) seedlings challenged for 0, 24, 48 or 96 h with 250 µM Zn. The location of the F1 and R1 primers used is shown in Figure 2A. Expression of the UBQ10 gene was used as a loading control. Results are representative of two independent experiments. (PDF) [file pgen.1004375.s001.pdf]

Figure S1

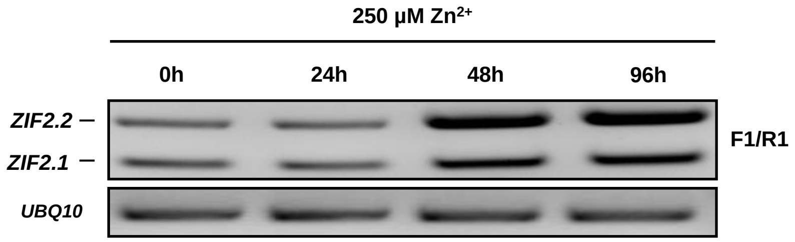

Figure S1 Expression kinetics of the *ZIF2.1* and *ZIF2.2* splice variants under zinc toxicity. RT-PCR profile of *ZIF2.1* and *ZIF2.2* expression in roots of 7-d old wild-type (Col-0) seedlings challenged for 0, 24, 48 or 96h with 250 µM Zn. The location of the F1 and R1 primers used is shown in Figure 2A. Expression of the *UBQ10* gene was used as a loading control. Results are representative of two independent experiments.
