# Supplementary material for: Intron Retention in the 5′UTR of the Novel ZIF2 Transporter Enhances Translation to Promote Zinc Tolerance in Arabidopsis
Source: PLoS Genet. 2014 May 15;10(5):e1004375. doi: 10.1371/journal.pgen.1004375 (PMC4022490; doi:10.1371/journal.pgen.1004375)
Supplement: Figure S2 — Heterologous expression of the Arabidopsis ZIF2 transporter in yeast. (A) Western blot analysis of the GFP-ZIF2 fusion protein in wild-type yeast cells harbouring either the cloning vector pGREG576 or the GFP-ZIF2-encoding pGREG576_ZIF2 plasmid after induction of recombinant protein production using anti-GFP antibodies. (B) Representative fluorescence microscopy images of wild-type yeast cells harbouring either the cloning vector pGREG576 (background fluorescence) or the GFP-ZIF2-encoding pGREG576_ZIF2 plasmid after induction of recombinant protein production, suggesting that the GFP-ZIF2 fusion protein is targeted to the yeast plasma membrane. Scale bars, 1 µm. (C) Comparison of the growth curves of non-adapted wild-type, Δzrt1zrt2 and Δzrc1cot1 mutant yeast cells, harbouring either the cloning vector pGREG576 or the GFP-ZIF2-encoding pGREG576_ZIF2 plasmid, in Zn-free liquid medium supplemented with 0.062 µM (wild-type and Δzrt1zrt2 strains), 2.5 or 20 µM (all strains), and 100, 250 or 500 µM (wild-type and Δzrc1cot1 strains) ZnSO4. Results are representative of three independent experiments (a replicate is shown in Figure 5). (PDF) [file pgen.1004375.s002.pdf]

Figure S2

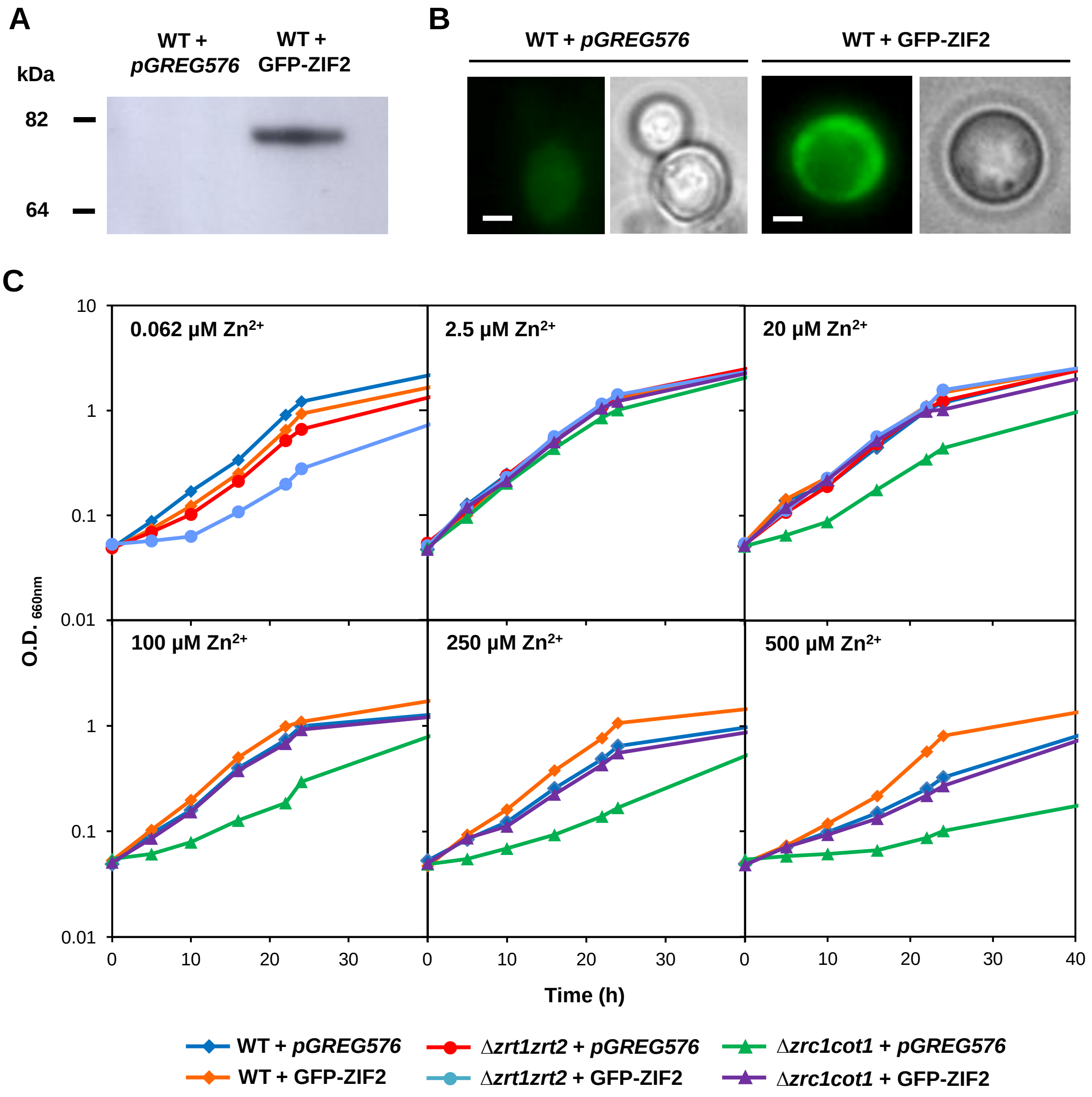

Figure S2 Heterologous expression of the *Arabidopsis* ZIF2 transporter in yeast. (**A**) Western blot analysis of the GFP-ZIF2 fusion protein in wild-type yeast cells harbouring either the cloning vector *pGREG576* or the GFP-ZIF2-encoding *pGREG576_ZIF2* plasmid after induction of recombinant protein production using anti-GFP antibodies. (**B**) Representative fluorescence microscopy images of wild-type yeast cells harbouring either the cloning vector *pGREG576* (background fluorescence) or the GFP-ZIF2-encoding *pGREG576_ZIF2* plasmid after induction of recombinant protein production, suggesting that the GFP-ZIF2 fusion protein is targeted to the yeast plasma membrane. Scale bars, 1 µm. (**C**) Comparison of the growth curves of non-adapted wild-type, Δ*zrt1zrt2* and Δ*zrc1cot1* mutant yeast cells, harbouring either the cloning vector *pGREG576* or the GFP-ZIF2-encoding *pGREG576_ZIF2* plasmid, in Zn-free liquid medium supplemented with 0.062 µM (wild-type and Δ*zrt1zrt2* strains), 2.5 or 20 µM (all strains), and 100, 250 or 500 µM (wild-type and Δ*zrc1cot1* strains) $ZnSO_4$. Results are representative of three independent experiments (a replicate is shown in Figure 5).
