# Supplementary material for: Intron Retention in the 5′UTR of the Novel ZIF2 Transporter Enhances Translation to Promote Zinc Tolerance in Arabidopsis
Source: PLoS Genet. 2014 May 15;10(5):e1004375. doi: 10.1371/journal.pgen.1004375 (PMC4022490; doi:10.1371/journal.pgen.1004375)
Supplement: Figure S3 — Further phenotypical characterisation of yeast cells expressing the Arabidopsis ZIF2 transporter. (A) Comparison of the growth curves of non-adapted wild-type and Δzrc1cot1 mutant yeast cells, harbouring either the cloning vector pGREG576 or the GFP-ZIF2-encoding pGREG576_ZIF2 plasmid, in liquid medium supplemented with 3.5 mM CoSO4, 1 mM NiSO4, 1 mM CuCl2, 40 mM FeSO4 or 25 mM MnCl2. Results are representative of at least two independent experiments. (B–C) Susceptibility to myo-inositol (B) and weak acids (C) of wild-type or Δitr1 mutant yeast cells, harbouring either the cloning vector pGREG576 or the GFP-ZIF2-encoding pGREG576_ZIF2 plasmid, determined by spotting dilution series of cell suspensions (1, 1∶5, and 1∶10). (PDF) [file pgen.1004375.s003.pdf]

Figure S3

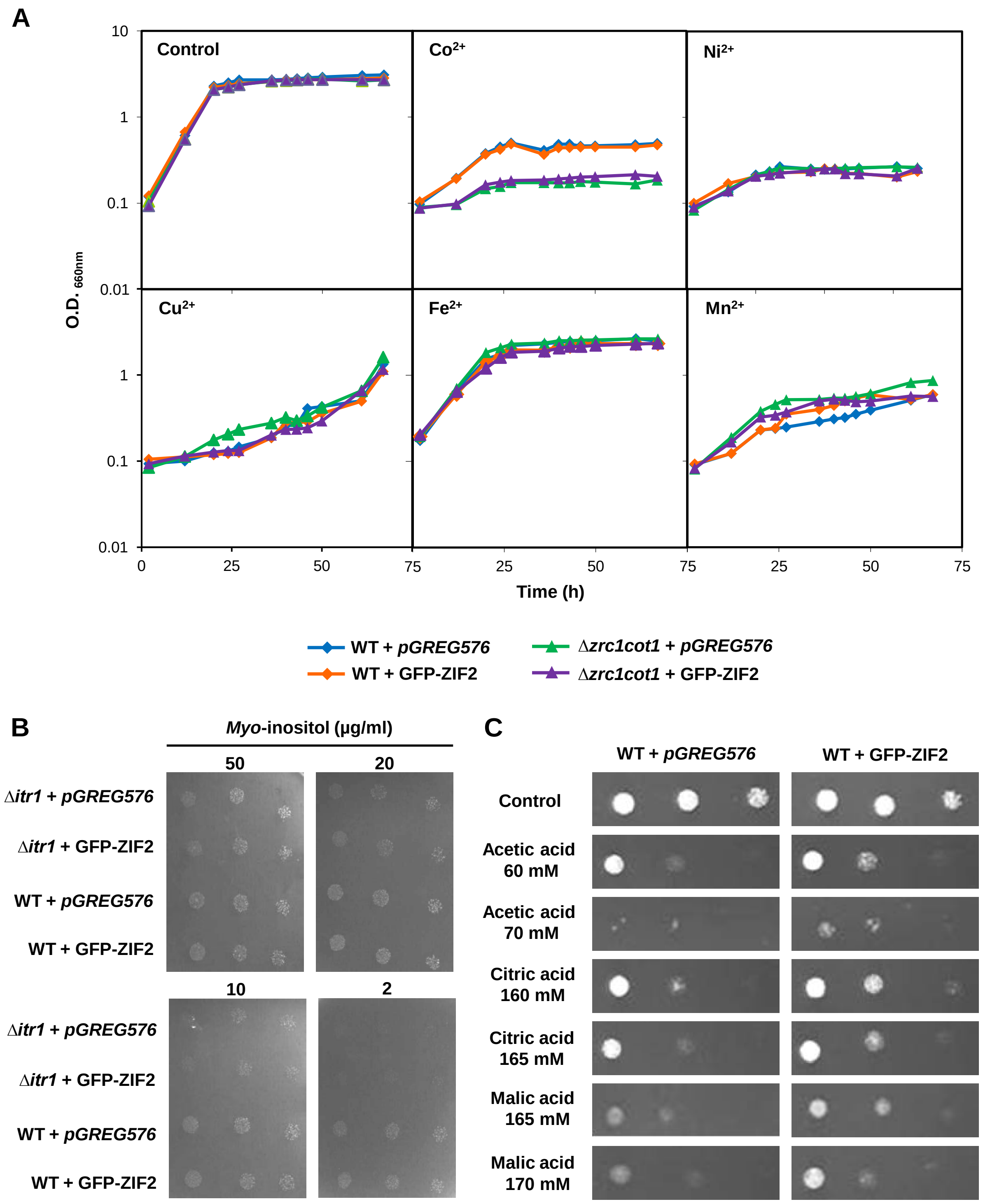

Figure S3 Further phenotypical characterisation of yeast cells expressing the *Arabidopsis* ZIF2 transporter. **(A)** Comparison of the growth curves of non-adapted wild-type and Δ*zrc1cot1* mutant yeast cells, harbouring either the cloning vector *pGREG576* or the GFP-ZIF2-encoding *pGREG576_ZIF2* plasmid, in liquid medium supplemented with 3.5 mM $CoSO_4$, 1 mM $NiSO_4$, 1 mM $CuCl_2$, 40 mM $FeSO_4$ or 25 mM $MnCl_2$. Results are representative of at least two independent experiments. **(B-C)** Susceptibility to *myo*-inositol **(B)** and weak acids **(C)** of wild-type or Δ*itr1* mutant yeast cells, harbouring either the cloning vector *pGREG576* or the GFP-ZIF2-encoding *pGREG576_ZIF2* plasmid, determined by spotting dilution series of cell suspensions (1, 1:5, and 1:10).
