# Supplementary material for: Intron Retention in the 5′UTR of the Novel ZIF2 Transporter Enhances Translation to Promote Zinc Tolerance in Arabidopsis
Source: PLoS Genet. 2014 May 15;10(5):e1004375. doi: 10.1371/journal.pgen.1004375 (PMC4022490; doi:10.1371/journal.pgen.1004375)
Supplement: Figure S4 — ZIF2 expression in the Arabidopsis zif2-1 mutant. RT-PCR analysis of ZIF2 expression in 14-d old wild-type (Col-0) and mutant (zif2-1) seedlings. The location of the F1′, R1, F2, R2, F3 and R3 primers used is shown in Figure 2A. Expression of the UBQ10 gene was used as a loading control. Results are representative of three independent experiments. (PDF) [file pgen.1004375.s004.pdf]

Figure S4

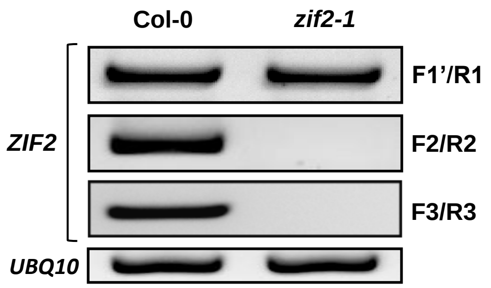

Figure S4 *ZIF2* expression in the *Arabidopsis zif2-1* mutant. RT-PCR analysis of *ZIF2* expression in 14-d old wild-type (Col-0) and mutant (*zif2-1*) seedlings. The location of the F1', R1, F2, R2, F3 and R3 primers used is shown in Figure 2A. Expression of the *UBQ10* gene was used as a loading control. Results are representative of three independent experiments.
