# Supplementary material for: Intron Retention in the 5′UTR of the Novel ZIF2 Transporter Enhances Translation to Promote Zinc Tolerance in Arabidopsis
Source: PLoS Genet. 2014 May 15;10(5):e1004375. doi: 10.1371/journal.pgen.1004375 (PMC4022490; doi:10.1371/journal.pgen.1004375)
Supplement: Figure S5 — Phenotype of the Arabidopsis zif2-1 mutant under zinc or iron deficiency. Effect of Zn deficiency under different MS strengths (A) and of ferrozine-induced Fe deficiency (B) on PR elongation of wild-type (Col-0) and zif2-1 or zif1-2 mutant seedlings. Results are representative of two independent experiments and values represent means ± SD (n = 16). Asterisks denote statistically significant differences from the wild type under each condition (***P<0.001; Student's t-test). (PDF) [file pgen.1004375.s005.pdf]

Figure S5

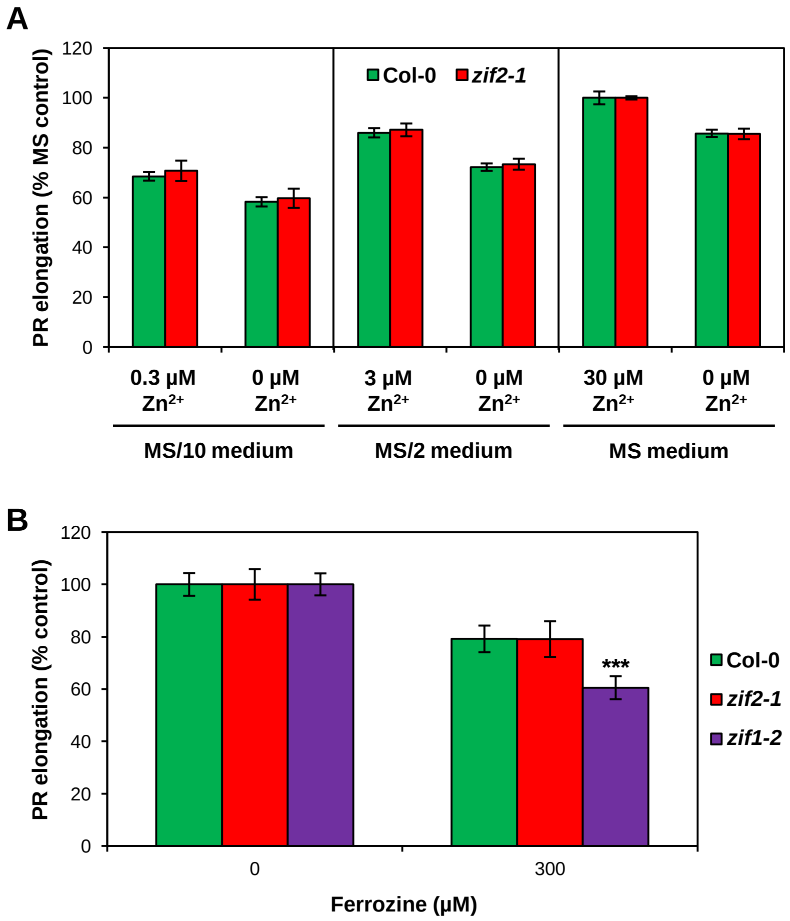

Figure S5 Phenotype of the *Arabidopsis zif2-1* mutant under zinc or iron deficiency. Effect of Zn deficiency under different MS strengths (**A**) and of ferrozine-induced Fe deficiency (**B**) on PR elongation of wild-type (Col-0) and *zif2-1* or *zif1-2* mutant seedlings. Results are representative of two independent experiments and values represent means ± SD (n=16). Asterisks denote statistically significant differences from the wild type under each condition (***P<0.001; Student's t-test).
