# Supplementary material for: Intron Retention in the 5′UTR of the Novel ZIF2 Transporter Enhances Translation to Promote Zinc Tolerance in Arabidopsis
Source: PLoS Genet. 2014 May 15;10(5):e1004375. doi: 10.1371/journal.pgen.1004375 (PMC4022490; doi:10.1371/journal.pgen.1004375)
Supplement: Figure S7 — Phenotypical characterisation of the Arabidopsis zif2-1 mutant in the presence of different concentrations of myo-inositol. Effect of myo-inositol on PR elongation of wild-type (Col-0) and mutant (zif2-1) seedlings. Results are representative of two independent experiments and values represent means ± SD (n = 16). No statistical differences between mutant and wild type were detected under each condition (P>0.05; Student's t-test). (PDF) [file pgen.1004375.s007.pdf]

Figure S7

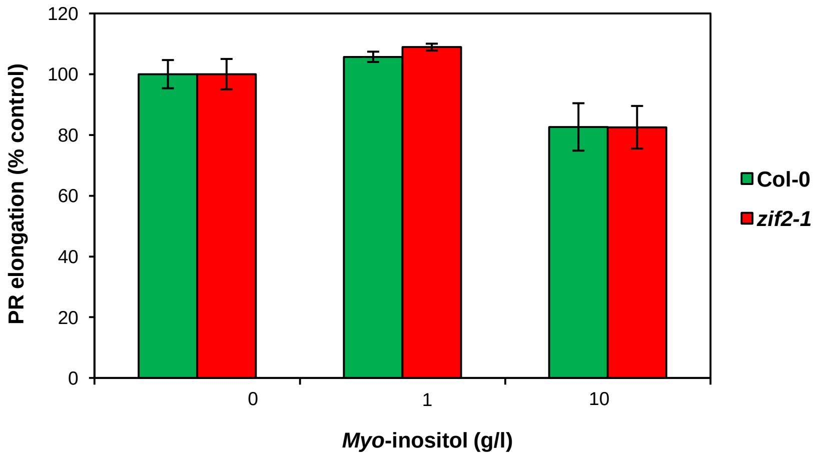

Figure S7 Phenotypical characterisation of the *Arabidopsis zif2-1* mutant in the presence of different concentrations of *myo*-inositol. Effect of *myo*-inositol on PR elongation of wild-type (Col-0) and mutant (*zif2-1)* seedlings. Results are representative of two independent experiments and values represent means ± SD (*n*=16). No statistical differences between mutant and wild type were detected under each condition (*P*>0.05; Student's *t*-test).
