# Supplementary material for: Intron Retention in the 5′UTR of the Novel ZIF2 Transporter Enhances Translation to Promote Zinc Tolerance in Arabidopsis
Source: PLoS Genet. 2014 May 15;10(5):e1004375. doi: 10.1371/journal.pgen.1004375 (PMC4022490; doi:10.1371/journal.pgen.1004375)
Supplement: Figure S8 — Phenotypical characterisation of the Arabidopsis zif2-1 mutant in the presence of both zinc and sugar. Effect of sucrose or glucose combined with Zn on shoot biomass (upper panel), chlorophyll content (middle panel) and PR elongation (lower panel) of wild-type (Col-0) and mutant (zif2-1) seedlings. Results are representative of two independent experiments and values represent means ± SD (n = 8 for shoot biomass/chlorophyll content and n = 16 for PR elongation). Asterisks denote statistically significant differences from the wild type under each condition (**P<0.01, ***P<0.001; Student's t-test). (PDF) [file pgen.1004375.s008.pdf]

# Figure S8

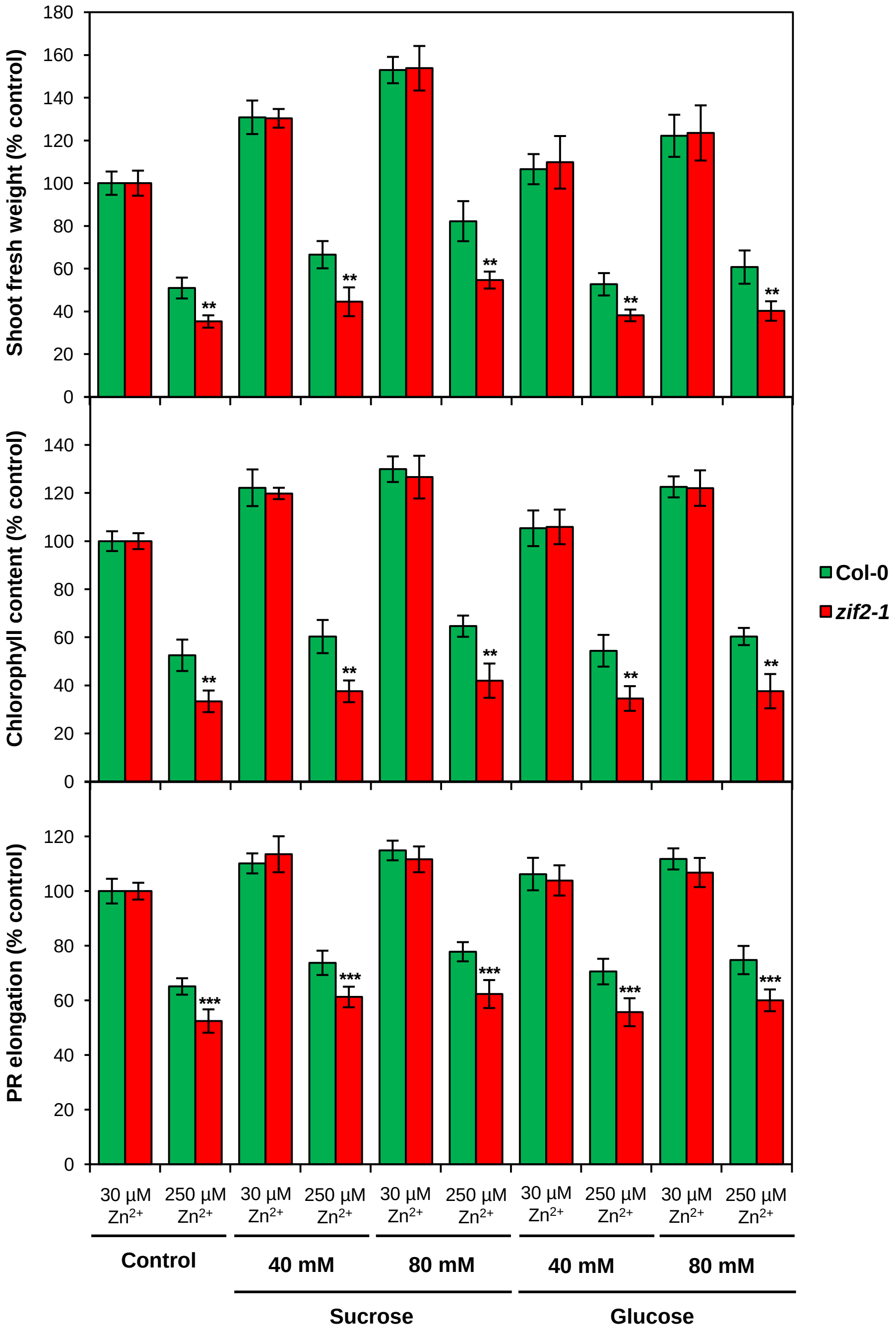


**Figure S8** Phenotypical characterisation of the *Arabidopsis zif2-1* mutant in the presence of both zinc and sugar. Effect of sucrose or glucose combined with Zn on shoot biomass (upper panel), chlorophyll content (middle panel) and PR elongation (lower panel) of wild-type (Col-0) and mutant (*zif2-1)* seedlings. Results are representative of two independent experiments and values represent means ± SD (*n*=8 for shoot biomass/chlorophyll content and *n*=16 for PR elongation). Asterisks denote statistically significant differences from the wild type under each condition (***P*<0.01, ****P*<0.001; Student's *t*-test).
