# Supplementary material for: Intron Retention in the 5′UTR of the Novel ZIF2 Transporter Enhances Translation to Promote Zinc Tolerance in Arabidopsis
Source: PLoS Genet. 2014 May 15;10(5):e1004375. doi: 10.1371/journal.pgen.1004375 (PMC4022490; doi:10.1371/journal.pgen.1004375)
Supplement: Figure S9 — Genomic complementation of the Arabidopsis zif2-1 mutant phenotype. Effect of Zn toxicity on shoot biomass (upper panel), chlorophyll content (middle panel) and PR elongation (lower panel) of seedlings of the wild type (Col-0), the zif2-1 mutant and three independent genomic complementation lines, zif2-1comp1-3. Results are representative of three independent experiments and values represent means ± SD (n = 8 for shoot biomass/chlorophyll content and n = 16 for PR elongation). Asterisks denote statistically significant differences from the wild type under each condition (*P<0.05, **P<0.01, ***P<0.001; Student's t-test). (PDF) [file pgen.1004375.s009.pdf]

Figure S9

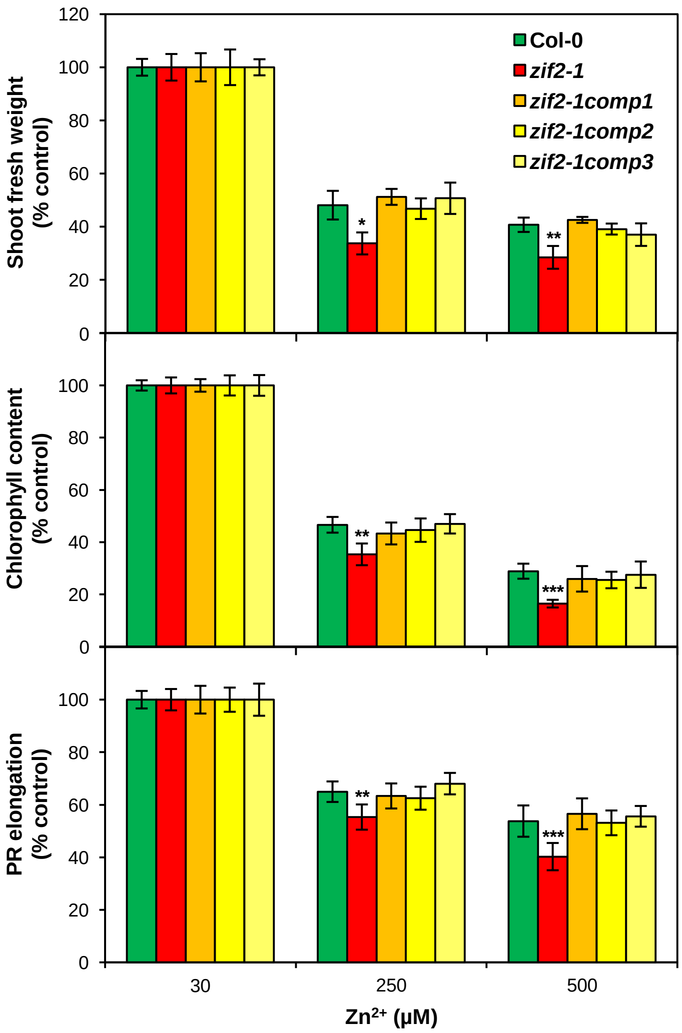

Figure S9 Genomic complementation of the *Arabidopsis zif2-1* mutant phenotype. Effect of Zn toxicity on shoot biomass (upper panel), chlorophyll content (middle panel) and PR elongation (lower panel) of seedlings of the wild type (Col-0), the *zif2-1* mutant and three independent genomic complementation lines, *zif2-1comp1-3.* Results are representative of three independent experiments and values represent means ± SD (n=8 for shoot biomass/chlorophyll content and n=16 for PR elongation). Asterisks denote statistically significant differences from the wild type under each condition (*P<0.05, **P<0.01, ***P<0.001; Student's t-test).
