# Supplementary material for: Intron Retention in the 5′UTR of the Novel ZIF2 Transporter Enhances Translation to Promote Zinc Tolerance in Arabidopsis
Source: PLoS Genet. 2014 May 15;10(5):e1004375. doi: 10.1371/journal.pgen.1004375 (PMC4022490; doi:10.1371/journal.pgen.1004375)
Supplement: Figure S10 — Phenotype of Arabidopsis ZIF2.1 and ZIF2.2 overexpression lines. Effect of Zn toxicity on shoot biomass (upper panel), chlorophyll content (middle panel) and PR elongation (lower panel) of wild-type (Col-0) and ZIF2.1- or ZIF2.2-overexpressing (ZIF2.1OX1-3 or ZIF2.2OX1-3) seedlings. Results are representative of three independent experiments and values represent means ± SD (n = 8 for shoot biomass/chlorophyll content and n = 16 for PR elongation). Different letters indicate statistically significant differences between genotypes under each condition (P<0.05; Student's t-test). (PDF) [file pgen.1004375.s010.pdf]

Figure S10

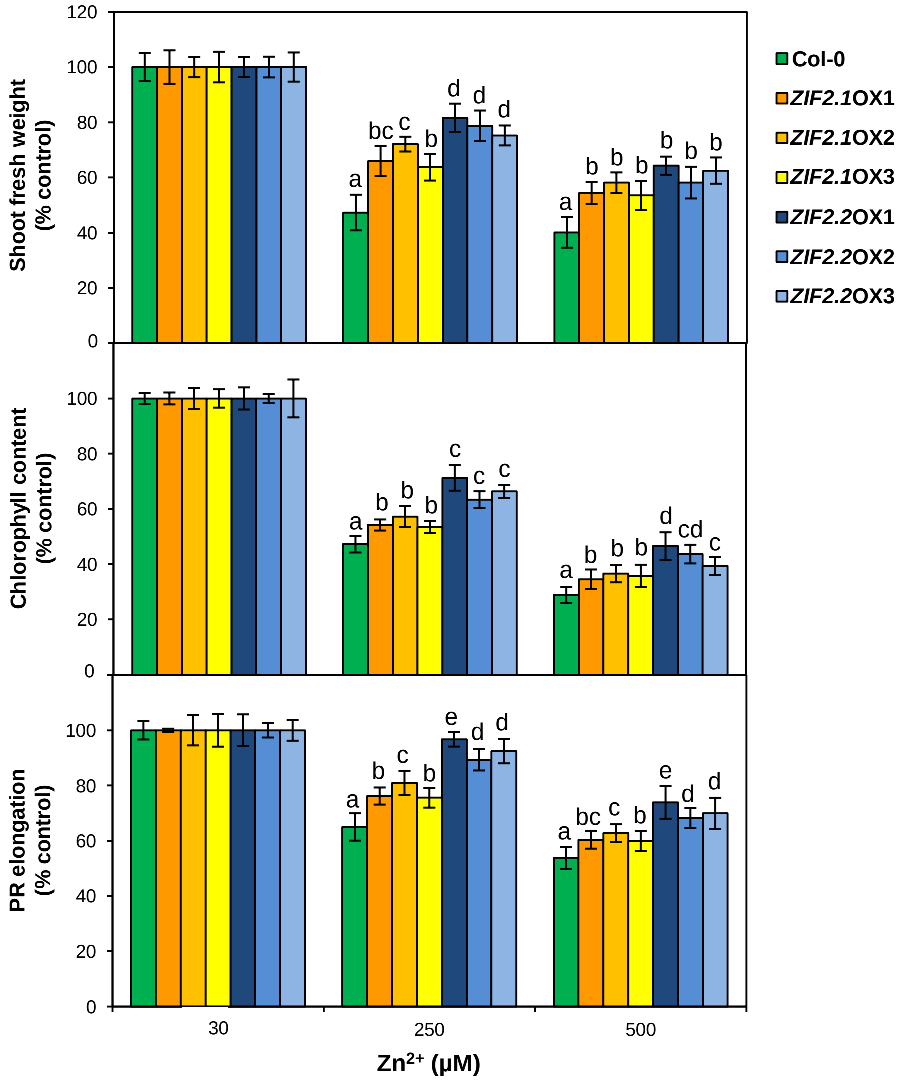

Figure S10 Phenotype of *Arabidopsis ZIF2.1* and *ZIF2.2* overexpression lines. Effect of Zn toxicity on shoot biomass (upper panel), chlorophyll content (middle panel) and PR elongation (lower panel) of wild-type (Col-0) and *ZIF2.1*- or *ZIF2.2*-overexpressing (*ZIF2.1*OX1-3 or *ZIF2.2*OX1-3) seedlings. Results are representative of three independent experiments and values represent means ± SD (n=8 for shoot biomass/chlorophyll content and n=16 for PR elongation). Different letters indicate statistically significant differences between genotypes under each condition (P<0.05; Student's t-test).
