# Supplementary material for: Intron Retention in the 5′UTR of the Novel ZIF2 Transporter Enhances Translation to Promote Zinc Tolerance in Arabidopsis
Source: PLoS Genet. 2014 May 15;10(5):e1004375. doi: 10.1371/journal.pgen.1004375 (PMC4022490; doi:10.1371/journal.pgen.1004375)
Supplement: Figure S11 — Whole-seedling zinc concentration of Arabidopsis wild-type, zif2-1 mutant and ZIF2 overexpression lines. Zn concentration, expressed on a dry weight (DW) basis, in 21-d old seedlings of the wild type (Col-0), the zif2-1 mutant and ZIF2-overexpressing lines (ZIF2.1OX2 and ZIF2.2OX1) grown on control medium (30 µM Zn2+) or under excess Zn supply (125 or 250 µM Zn2+). Bars represent means ± SD (n = 4). No statistical differences between genotypes were detected under each condition (P>0.05; Student's t-test). (PDF) [file pgen.1004375.s011.pdf]

Figure S11

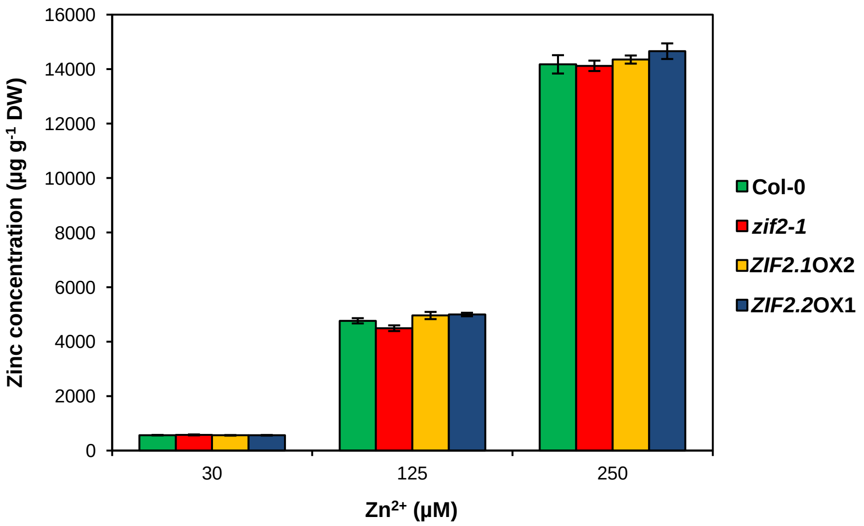

Figure S11 Whole-seedling zinc concentration of *Arabidopsis* wild-type, *zif2-1* mutant and *ZIF2* overexpression lines. Zn concentration, expressed on a dry weight (DW) basis, in 21-d old seedlings of the wild type (Col-0), the *zif2-1* mutant and *ZIF2*-overexpressing lines (*ZIF2.1*OX2 and *ZIF2.2*OX1) grown on control medium (30 µM Zn^{2+}) or under excess Zn supply (125 or 250 µM Zn^{2+}). Bars represent means ± SD (n=4). No statistical differences between genotypes were detected under each condition (P>0.05; Student's t-test).
