# Supplementary material for: Intron Retention in the 5′UTR of the Novel ZIF2 Transporter Enhances Translation to Promote Zinc Tolerance in Arabidopsis
Source: PLoS Genet. 2014 May 15;10(5):e1004375. doi: 10.1371/journal.pgen.1004375 (PMC4022490; doi:10.1371/journal.pgen.1004375)
Supplement: Figure S12 — Phenotype of Arabidopsis ZIF2.1-YFP and ZIF2.2-YFP overexpression lines. Effect of Zn toxicity on PR elongation of wild type (Col-0) and ZIF2.1-YFP or ZIF2.2-YFP overexpression lines (ZIF2.1-YFPOX1-2 or ZIF2.2-YFPOX1-2) seedlings. Results are representative of two independent experiments and values represent means ± SD (n = 16). Different letters indicate statistically significant differences between genotypes under each condition (P<0.05; Student's t-test). (PDF) [file pgen.1004375.s012.pdf]

Figure S12

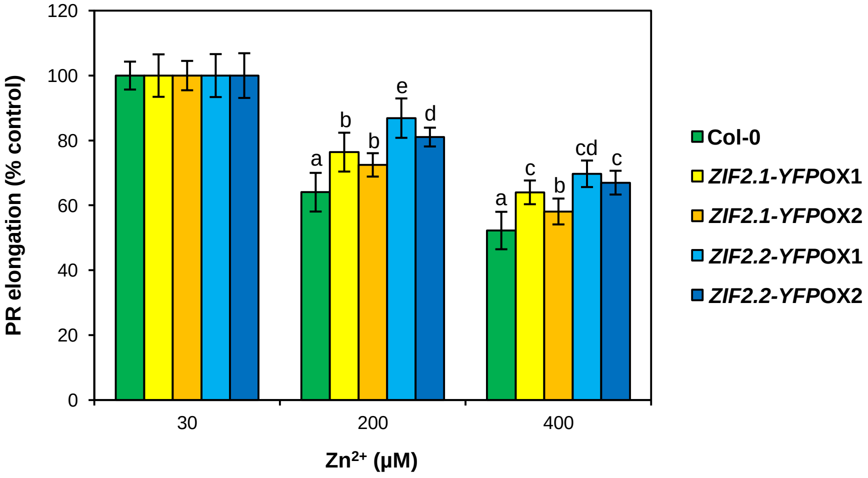

Figure S12 Phenotype of *Arabidopsis ZIF2.1-YFP* and *ZIF2.2-YFP* overexpression lines. Effect of Zn toxicity on PR elongation of wild type (Col-0) and *ZIF2.1-YFP* or *ZIF2.2-YFP* overexpression lines (*ZIF2.1-YFP*OX1-2 or *ZIF2.2-YFP*OX1-2) seedlings. Results are representative of two independent experiments and values represent means ± SD (n=16). Different letters indicate statistically significant differences between genotypes under each condition (P<0.05; Student's t-test).
