# Supplementary material for: Intron Retention in the 5′UTR of the Novel ZIF2 Transporter Enhances Translation to Promote Zinc Tolerance in Arabidopsis
Source: PLoS Genet. 2014 May 15;10(5):e1004375. doi: 10.1371/journal.pgen.1004375 (PMC4022490; doi:10.1371/journal.pgen.1004375)
Supplement: Figure S13 — Quantification of ZIF2-YFP protein levels in Arabidopsis ZIF2-YFP overexpression lines. Quantification of the YFP signal by confocal laser scanning microscopy in primary root tip (upper panel) and primary root (lower panel) cells from 5-d old seedlings of the ZIF2.1-YFPOX and ZIF2.2-YFPOX transgenic lines. In each panel, detection settings for YFP visualization were identical for all genotypes. Results are representative of three (upper panel) or two (lower panel) independent experiments and average fluorescence (pixel) intensity is shown (means ± SD, n = 12). Asterisks denote statistically significant differences from the ZIF2.1-YFPOX1 line (***P<0.001; Student's t test). (PDF) [file pgen.1004375.s013.pdf]

Figure S13

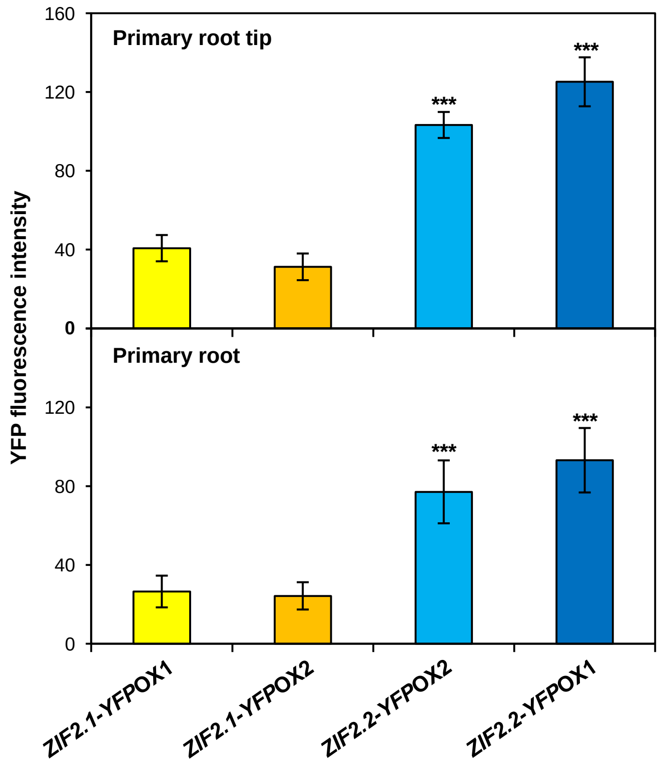

Figure S13 Quantification of ZIF2-YFP protein levels in *Arabidopsis ZIF2-YFP* overexpression lines. Quantification of the YFP signal by confocal laser scanning microscopy in primary root tip (upper panel) and primary root (lower panel) cells from 5-d old seedlings of the *ZIF2.1-YFP*OX and *ZIF2.2-YFP*OX transgenic lines. In each panel, detection settings for YFP visualization were identical for all genotypes. Results are representative of three (upper panel) or two (lower panel) independent experiments and average fluorescence (pixel) intensity is shown (means ± SD, n=12). Asterisks denote statistically significant differences from the *ZIF2.1-YFP*OX1 line (***P<0.001; Student's t test).
