# Supplementary material for: Intron Retention in the 5′UTR of the Novel ZIF2 Transporter Enhances Translation to Promote Zinc Tolerance in Arabidopsis
Source: PLoS Genet. 2014 May 15;10(5):e1004375. doi: 10.1371/journal.pgen.1004375 (PMC4022490; doi:10.1371/journal.pgen.1004375)
Supplement: Figure S14 — Effect of zinc on transgene mRNA expression and luciferase (LUC) activity driven by the 35S promoter. (A) Real-time RT-PCR analysis of ZIF2.15′UTR-LUC and ZIF2.25′UTR-LUC transcript levels in protoplasts transiently expressing the Pro35S:ZIF2.15′UTR-LUC (orange bars) or Pro35:ZIF2.25′UTR-LUC (blue bars) constructs under various Zn supplies, using UBQ10 as a reference gene. Results are from two independent experiments and values represent means ± SE (n = 4). No statistical differences were detected for each transcript between the different conditions (P>0.05; Student's t-test). (B) Protoplast transient expression assay using the Pro35S:LUC (reference control) and Pro35S:GUSmutated (negative control, no detectable LUC activity) constructs under various Zn supplies. Results are representative of three independent experiments, corresponding to the same assay shown in Figure 9C. Values represent means ± SD (n = 4). P values inside the bars indicate statistical differences for the Pro35S:LUC construct (comparison with 0 µM Zn condition) obtained by Student's t-test. (PDF) [file pgen.1004375.s014.pdf]

# Figure S14

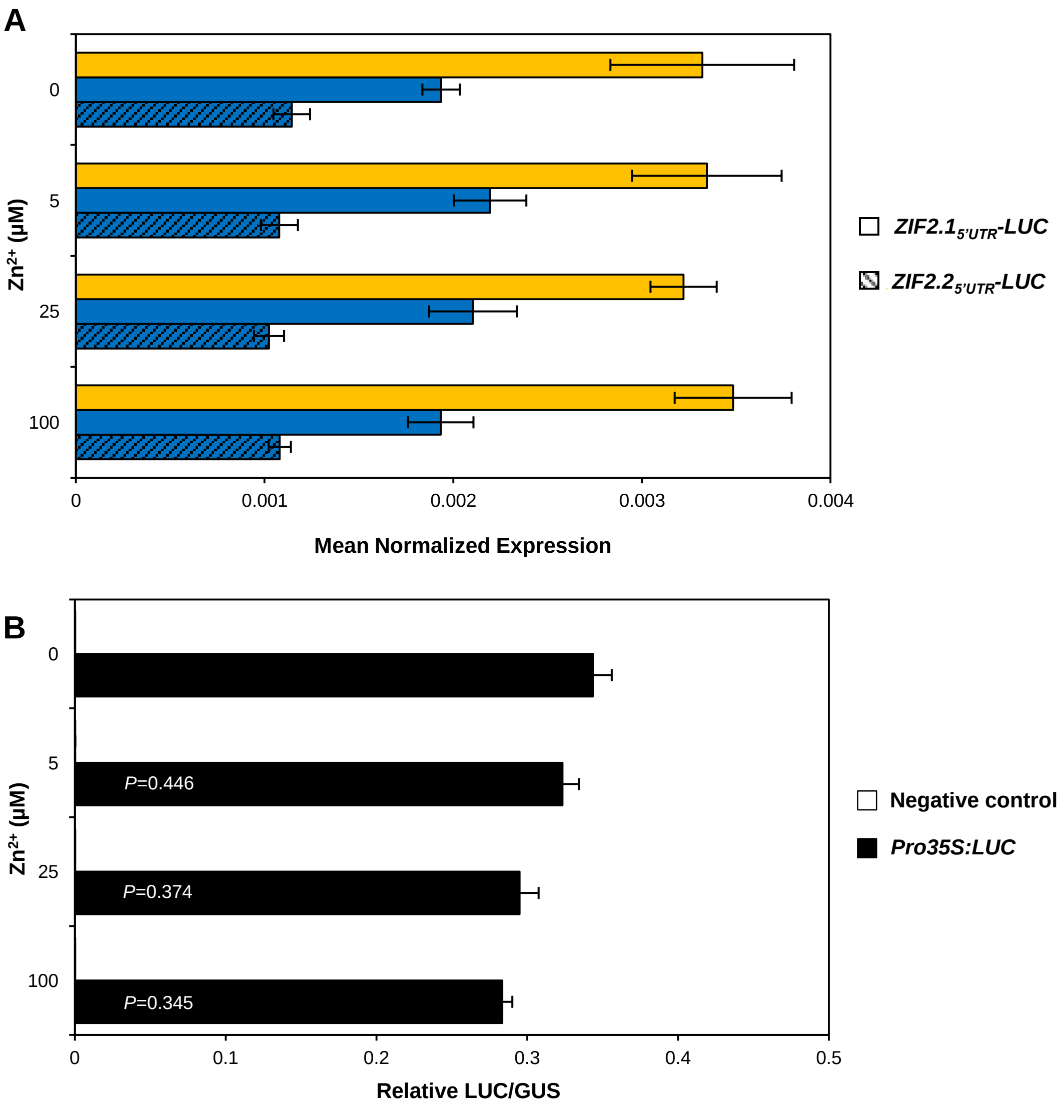


**Figure S14** Effect of zinc on transgene mRNA expression and luciferase (LUC) activity driven by the 35S promoter. (**A**) Real-time RT-PCR analysis of *ZIF2.1*$_{5'UTR}$*-LUC* and *ZIF2.2*$_{5'UTR}$*-LUC* transcript levels in protoplasts transiently expressing the *Pro35S:ZIF2.1*$_{5'UTR}$*-LUC* (orange bars) or *Pro35:ZIF2.2*$_{5'UTR}$*-LUC* (blue bars) constructs under various Zn supplies, using *UBQ10* as a reference gene. Results are from two independent experiments and values represent means ± SE (*n*=4). No statistical differences were detected for each transcript between the different conditions (*P*>0.05; Student's *t*-test). (**B**) Protoplast transient expression assay using the *Pro35S:LUC* (reference control) and *Pro35S*:GUS$_{mutated}$ (negative control, no detectable LUC activity) constructs under various Zn supplies. Results are representative of three independent experiments, corresponding to the same assay shown in Figure 9C. Values represent means ± SD (*n*=4). *P* values inside the bars indicate statistical differences for the *Pro35S:LUC* construct (comparison with 0 µM Zn condition) obtained by Student's *t*-test.
