# Supplementary material for: Intron Retention in the 5′UTR of the Novel ZIF2 Transporter Enhances Translation to Promote Zinc Tolerance in Arabidopsis
Source: PLoS Genet. 2014 May 15;10(5):e1004375. doi: 10.1371/journal.pgen.1004375 (PMC4022490; doi:10.1371/journal.pgen.1004375)
Supplement: Table S1 — Growth parameters of Arabidopsis ZIF2 loss-of-function and overexpression lines under control conditions. (PDF) [file pgen.1004375.s015.pdf]

Table S1 Growth parameters of *Arabidopsis ZIF2* loss-of-function and overexpression lines under control conditions.

	Shoot parameters		Root parameters		
Genotype	FW (mg/plant)	CC (mg/mg plant)	PRE (cm)	LRD (LR/cm)	TLRL (cm)
Col-0	9.04 ± 0.99	1.11 ± 0.30	3.97 ± 0.43	2.65 ± 0.35	9.75 ± 1.28
zif2-1	8.96 ± 1.55 (0.457)	1.08 ± 0.16 (0.404)	3.94 ± 0.59 (0.465)	2.67 ± 0.39 (0.406)	9.87 ± 4.31 (0.461)
*ZIF2.1*OX1	8.60 ± 1.39 (0.254)	0.97 ± 0.20 (0.165)	3.95 ± 0.77 (0.482)	2.51 ± 0.21 (0.082)	9.24 ± 1.35 (0.150)
*ZIF2.1*OX2	8.60 ± 1.71 (0.283)	1.03 ± 0.19 (0.295)	3.69 ± 0.59 (0.085)	2.50 ± 0.54 (0.177)	9.46 ± 4.83 (0.412)
*ZIF2.1*OX3	8.90 ± 2.24 (0.442)	1.10 ± 0.24 (0.474)	4.07 ± 0.60 (0.279)	2.49 ± 0.47 (0.145)	9.47 ± 3.99 (0.398)
*ZIF2.2*OX1	8.83 ± 2.24 (0.411)	0.93 ± 0.26 (0.121)	3.74 ± 0.42 (0.090)	2.60 ± 0.65 (0.401)	9.86 ± 4.06 (0.459)
*ZIF2.2*OX2	8.80 ± 2.14 (0.397)	0.92 ± 0.12 (0.076)	3.79 ± 0.34 (0.122)	2.54 ± 0.73 (0.298)	9.12 ± 2.50 (0.195)
*ZIF2.2*OX3	8.90 ± 2.24 (0.442)	1.03 ± 0.19 (0.288)	3.71 ± 0.54 (0.086)	2.53 ± 0.53 (0.234)	9.49 ± 2.76 (0.370)

Shoot biomass (fresh weight, FW), chlorophyll content (CC), PR elongation (PRE), lateral root density (LRD) and total lateral root length (TLRL) of 7-d (PRE) or 14-d (FW, CC, LRD, TLRL) old seedlings from the wild type (Col-0), the *zif2-1* mutant or *ZIF2.1*- and *ZIF2.2*-overexpressing lines grown on medium containing 30 µM Zn^{2+}. Results are representative of three independent experiments and values represent means ± SD (n=8 for FW/CC and n=16 for PRE, LRD and TLRL). Numbers between parentheses indicate the P value (comparison with the wild type) obtained by Student's t-test.
