# Supplementary material for: Intron Retention in the 5′UTR of the Novel ZIF2 Transporter Enhances Translation to Promote Zinc Tolerance in Arabidopsis
Source: PLoS Genet. 2014 May 15;10(5):e1004375. doi: 10.1371/journal.pgen.1004375 (PMC4022490; doi:10.1371/journal.pgen.1004375)
Supplement: Table S2 — Sequences of primers used. (PDF) [file pgen.1004375.s016.pdf]

Table S2 Sequences of primers used.

PRIMER NAME	SEQUENCE (5' to 3')
**RLM-5'RACE**	
RACE1	CACAAGAACCGAACGCAAAA
RACE2	GAACCACAGACCGCAACGAA
RACE3	CACGGCTGGTCTGGTTTTGA
RACE4	TGGTTCTCTGACGGCATCAC
RACE5	GAGACGAAGCCATTCAAGAG
RACE6	GAAAAGCGGAGGACAAGAAT
**Expression analyses**	
Semi-quantitative RT-PCR	
F1	CCGAGTGGGCTCAACG
F1'	CAAGTGATGCCGTCAGAGAA
F2	CGCTGCCTCCTTCCTT
F3	AGCAGCGGTTTCTTTCTATC
R1	GCCACCCGACTACACAAAA
R2	AGAAACCGCTGCTATCAAAC
R3	TGGCAGCGTAAATGAGG
ROC1-F	GTCTGATAGAGATCTCACGT
ROC1-R	AATCGGCAACAACAACAGGC
UBQ10-F1	GATCTTTGCCGGAAAACAATTGG
UBQ10-R1	TAGAAAGAAAGAGATAACAGG
Real-time RT-PCR	
ZIF2.1-F	CCCGAGTGGGCTCAA
ZIF2.1-R	GAATGGCTTCGTCTCTTT
ZIF2.2-F	TTGGTAAAGGACATAAAAC
ZIF2.2-R	GAAAAGCGGAGGACAAGAAT
ZIF2-F	AGAAAGGGGGCGATGAGAGT
ZIF2-R	CCAGTCTTCCGAGGTCCAG
UBQ10-F2	GACAATCACCCTCGAGGTGG
UBQ10-R2	CATCCTCTAGCTGCTTGCCG
ZIF2.1-LUC-F	ATCTCTTGAATGGCTTCG
ZIF2.1-LUC-R	CAGCGGTTCCATCCT
ZIF2.2-LUC-F	CGCTTTTCCATTTTACTCTT
ZIF2.2-LUC-R	GGGCCTTTCTTTATGTTTTT
**Cloning of Pro*ZIF2:GUS-GFP* construct**	
ProZIF2-F	TT*GAGCTC*GACCGCTCTGTTAGT
ProZIF2-R	TT*CCGCGG*CTTCAGGTATTATGTATCTC
**Cloning of Pro*ZIF2:ZIF2* construct**	
ZIF2comp-F	TT*CCATGG*GACCGCTCTGTTAGT
ZIF2comp-R	TT*TCTAGA*ACTATGACTCGGGTTTCTAT
**Cloning of Pro*35S:ZIF2-YFP* and Pro*35S:ZIF2-GFP* constructs**	
ZIF2YFP-F	TT*CTCGAG*CCGAGTGGGCTCAACG
ZIF2YFP-R	TT*AATTAA*TGGATTGACTATAGCTTGGA
**Cloning of Pro*35S:ZIF2* constructs**	
ZIF2OX-F	TT*CTCGAG*CCGAGTGGGCTCAACG
ZIF2OX-R	TT*GGCGCGCC*TGGATTGACTATAGCTTGGA
**Cloning of Pro*35S:ZIF2*$_{5'UTR}$*-LUC* constructs**	
ZIF2UTR-F	TT*GTCGAC*CCGAGTGGGCTCAACG
ZIF2UTR-R	TTT*CATGAC*TTCAGGTATTATGTATCTC
ZIF2UTRmutM-F	TATGTTTCTGTCTTCTTAAGTCACTTTTGCTTCAGGCTTC
ZIF2UTRmutM-R	GAAGCCTGAAGCAAAAGTGACTTAAGAAGACAGAAACATA
ZIF2UTRmutR-F	CGTCTCTTTTGCAATTTTGACTTTAGCCAAGTGCTCAAGG
ZIF2UTRmutR-R	CCTTGAGCACTTGGCTAAAGTCAAAATTGCAAAAGAGACG

Restriction sites are shown in italics and underlined.
